# Supplementary material for: p107 mediated mitochondrial function controls muscle stem cell proliferative fates
Source: Nat Commun. 2021 Oct 13;12:5977. doi: 10.1038/s41467-021-26176-0 (PMC8514468; doi:10.1038/s41467-021-26176-0)
Supplement: Supplementary file 1 — Supplementary Information [file 41467_2021_26176_MOESM1_ESM.pdf]

p107 mediated mitochondrial function controls muscle stem cell proliferative fates

Debasmita Bhattacharya[1,2], Vicky Shah[1,3], Oreoluwa Oresajo[1,2] and Anthony Scimè[1,2,3*]

1. Stem Cell Research Group

2. Molecular, Cellular and Integrative Physiology

Faculty of Health, York University,

3. Department of Biology

York University

Toronto, Ontario

M3J 1P3, Canada

* Corresponding author E mail: ascime@yorku.ca

Tel: 1 416 736 2100 ext 33559

Supplemental Figure 1

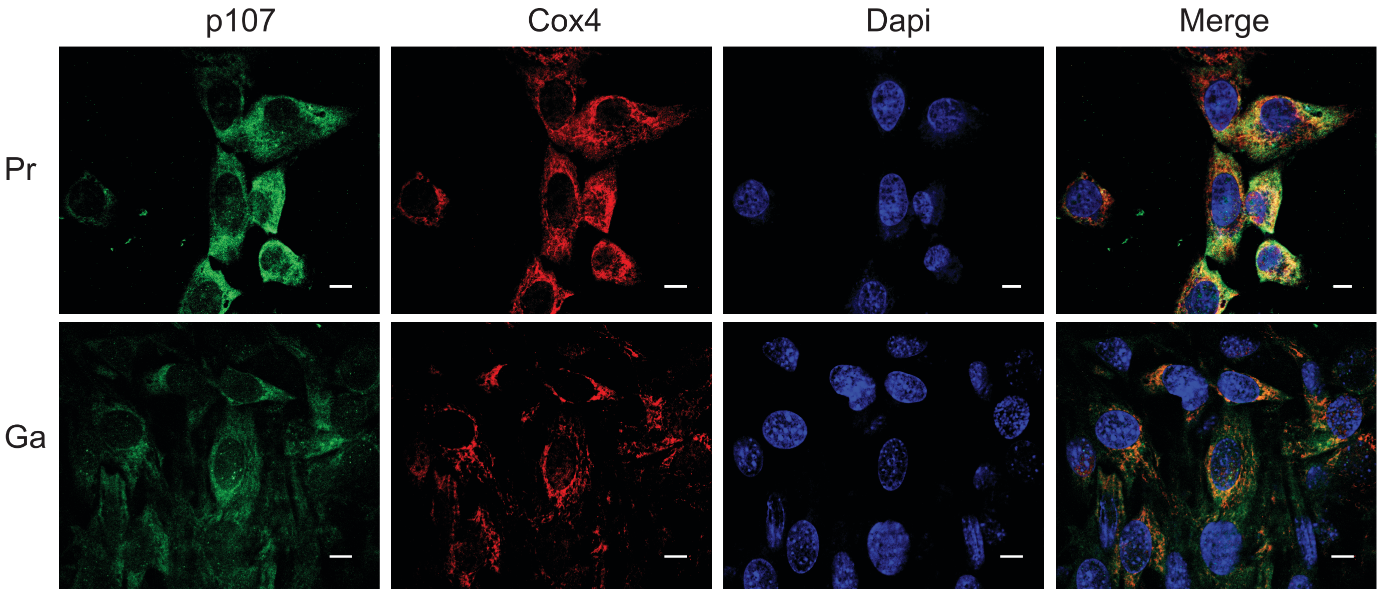

Suppl. Fig. 1 Reduced p107 colocalization with Cox4 in growth arrested cells. Confocal immunofluorescence microscopy for p107, Cox4, Dapi and Merge of proliferating (Pr) and growth arrested (Ga) c2MPs (scale bar 10μm).

Supplemental Figure 2

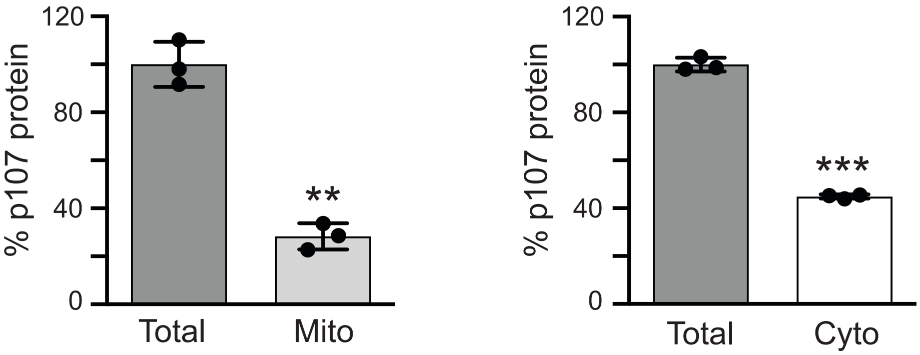

Suppl. Fig. 2 Percentage of p107 protein that is localized in the mitochondria and cytoplasm. Percentage determination from the total cellular p107 protein levels in mitochondria and cytoplasm. Data are presented as mean values ± SD (n = 3 biologically independent samples). Two-tailed unpaired Student T-test, $**p = 0.001$, $***p < 0.001$.

Supplemental Figure 3

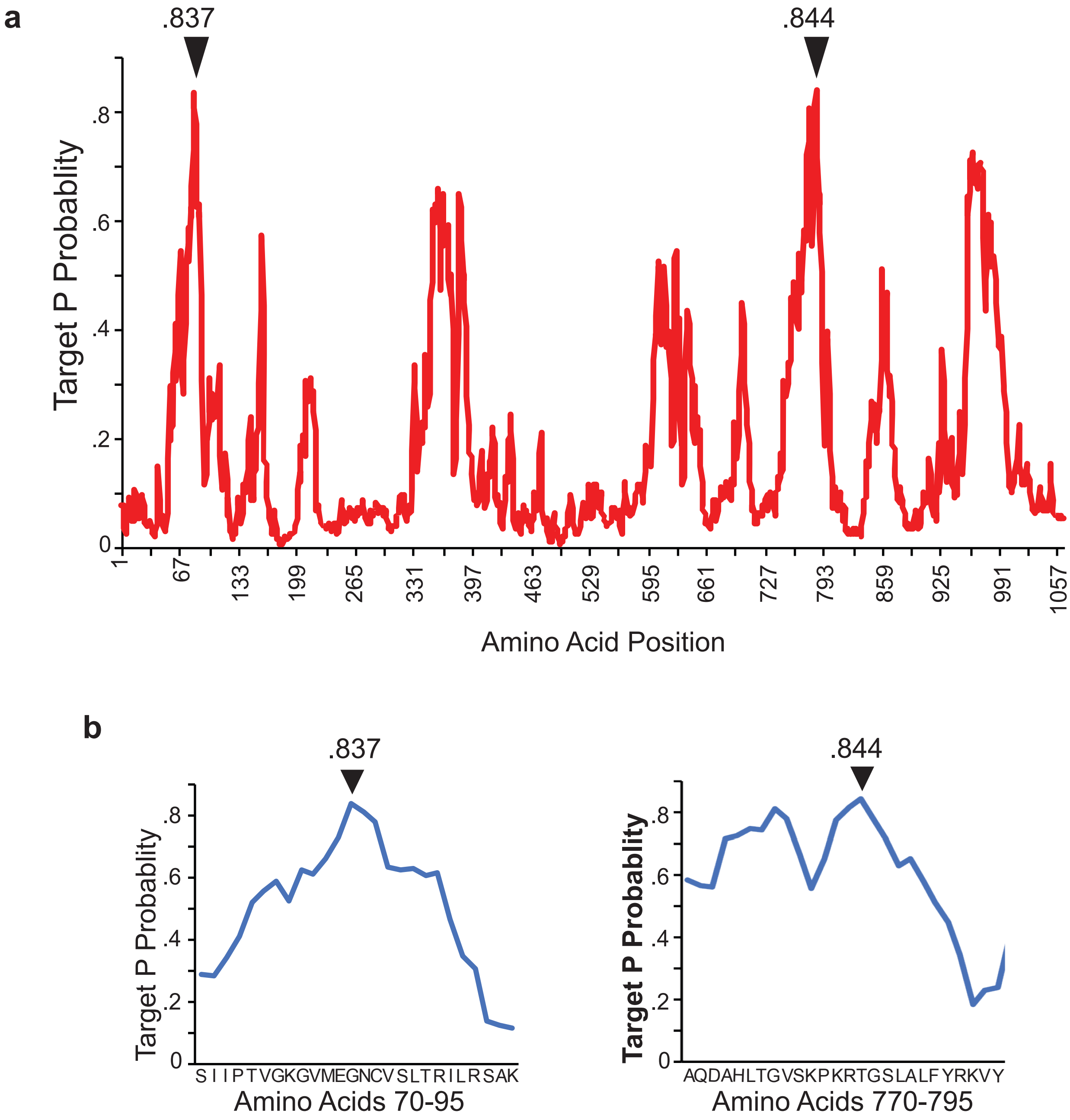

Suppl. Fig. 3 p107 has potential internal mitochondrial targeting signals (mts). a Internal mts-like TargetP probability scores for mouse p107 at amino acid positions. **b** Internal mts-like TargetP probability scores for p107 at amino acids 70-95 and 770-795. Arrows denote the highest probability scores of .837 and .844 (>0.75 is a strong predictor for localization).

Supplemental Figure 4

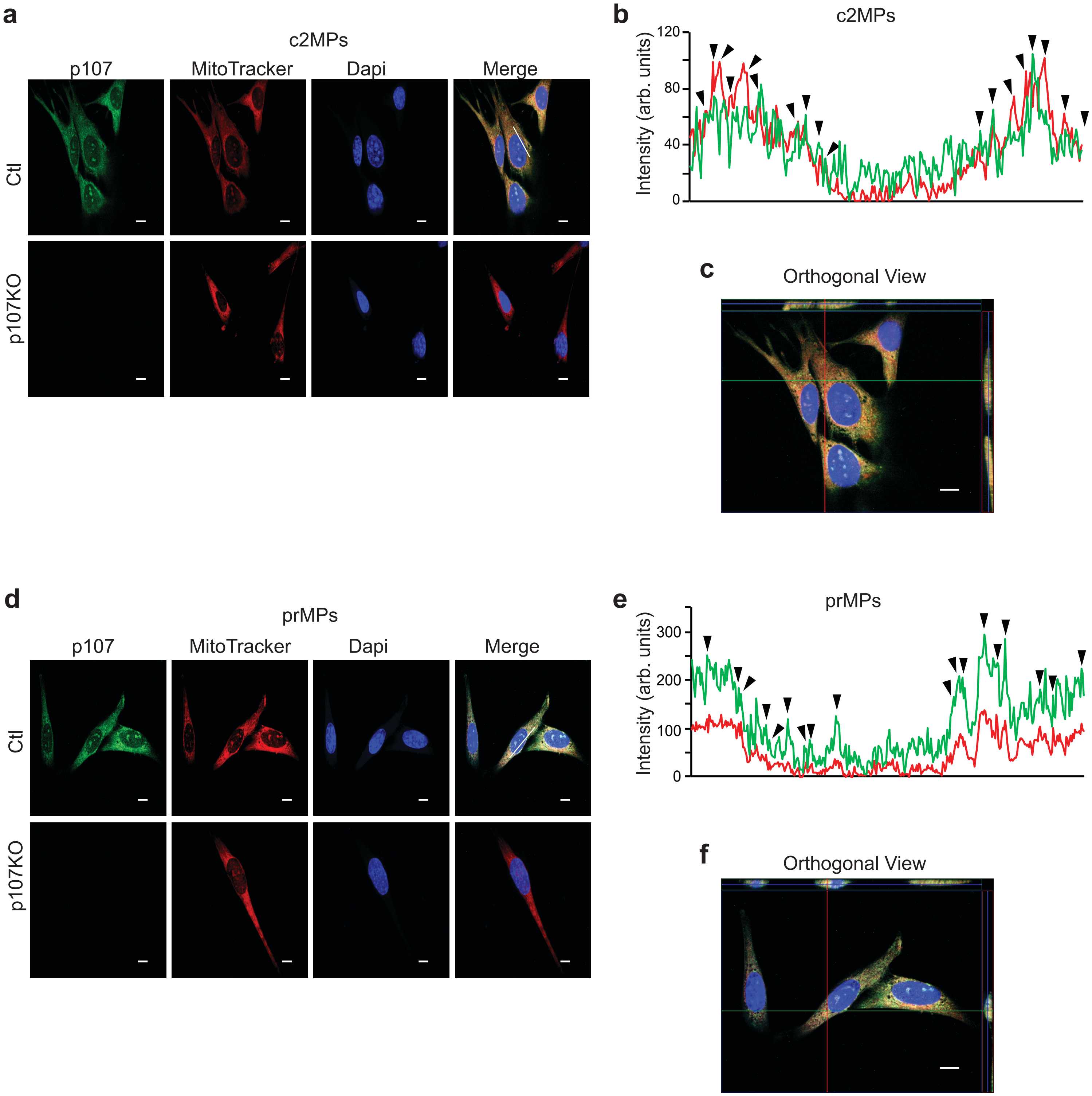

Suppl. Fig. 4 p107 colocalizes with MitoTracker red in proliferating myogenic progenitor cells. Confocal immunofluorescence microscopy for p107, MitoTracker red, Dapi and Merge of proliferating **a** Control (Ctl) and genetically deleted p107 (p107KO) c2MPs and **d** wild type (Wt) and p107KO primary (pr) MPs (scale bar 10µm). **b** and **e** A line was drawn through a representative cell to indicate relative intensity of RGB signals with the arrowheads pointing to areas of concurrent intensities **c** and **f** an orthogonal projection was generated by a Z-stack (100 nm interval) image set using the ZEN program (Zeiss) in the XY, XZ, and YZ planes (scale bar 10µm).

Supplemental Figure 5

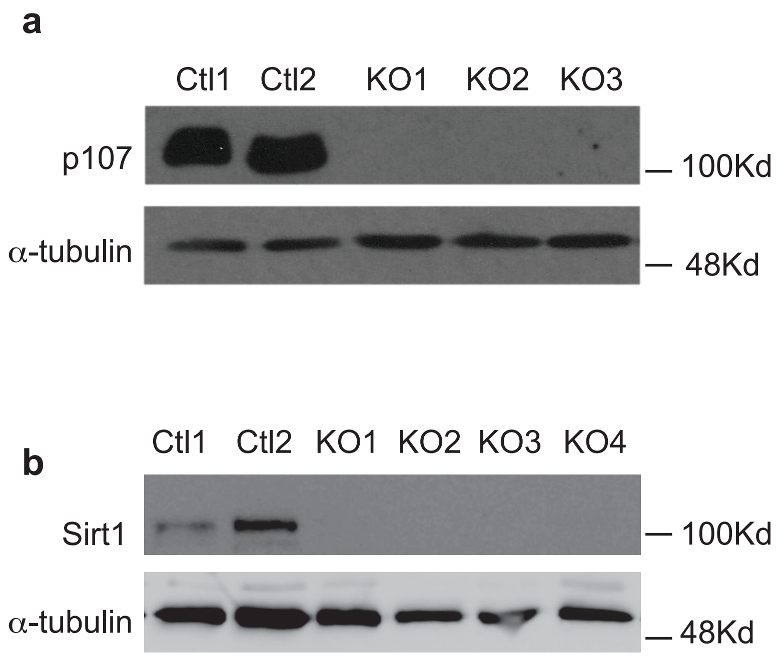

Suppl. Fig. 5 Crispr/Cas9 generated p107 and Sirt1 genetically deleted c2MPs. Representative Western blot for p107, Sirt1 and α-tubulin of different control c2MPs (Ctl) and **a** p107 or **b** Sirt1 genetically deleted c2MPs (KO).

Supplemental Figure 6

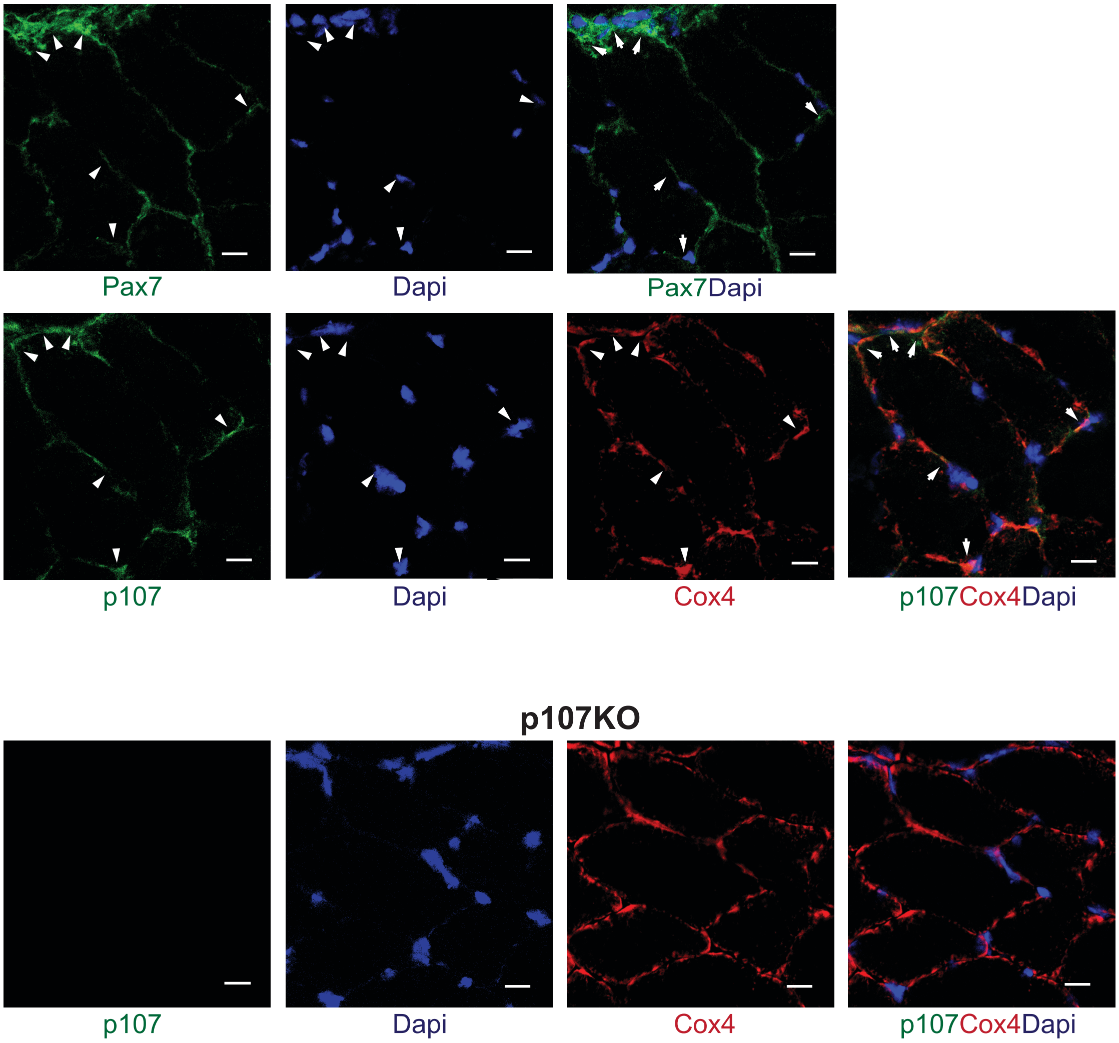

Suppl. Fig. 6 Individual fluorescence channels corresponding to images in Figure 1l. Confocal immunofluorescence microscopic image of wild type (Wt) tibialis anterior (TA) muscle section 2 days post injury for Pax7 (green), Dapi (blue), Merge and p107 (green), Dapi (blue), Cox4 (red), Merge and of p107KO TA muscle section for p107 (green), Dapi (blue), Cox4 (red), Merge as a negative control. Arrows denote Pax7^{+}p107^{+}Cox4^{+} cells (scale bar 20μm).

Supplemental Figure 7

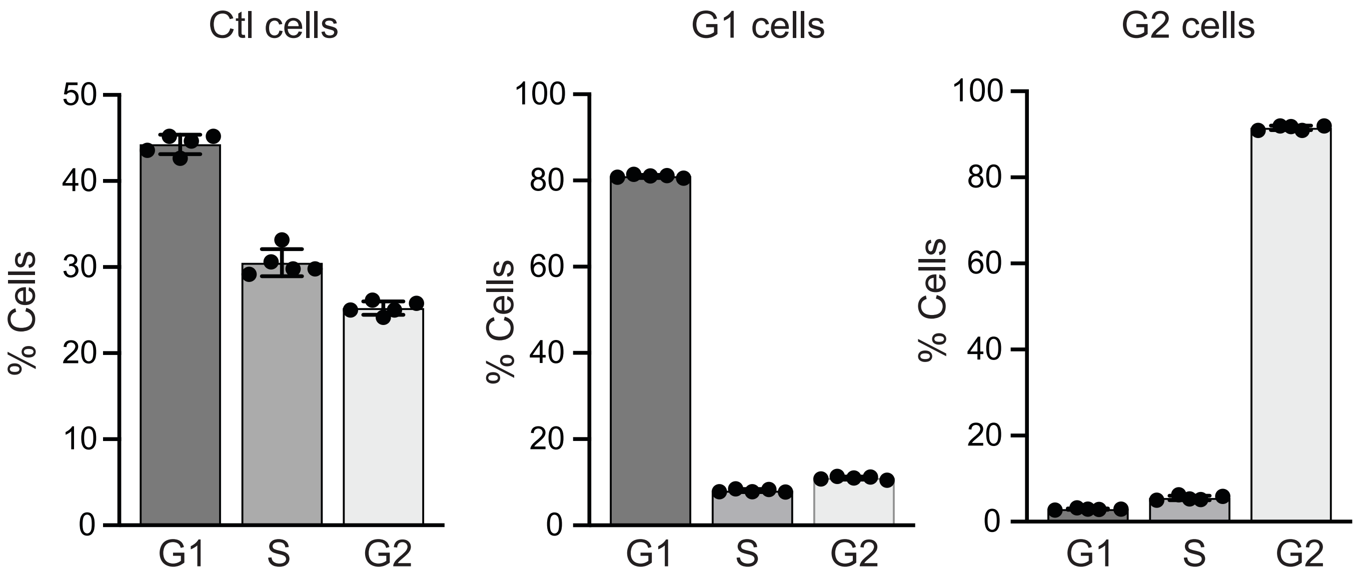

Suppl. Fig. 7 Cell cycle profile for cells that are asynchronous proliferating (Ctl cells), predominately in the G1 phase by serum starvation (G1 cells), and in the G2 phase by nocadazole treatment (G2 cells). Data are presented as mean values ± SD (n = 5 biologically independent samples). Data analyzed by two tailed unpaired Student T-test.

Supplemental Figure 8

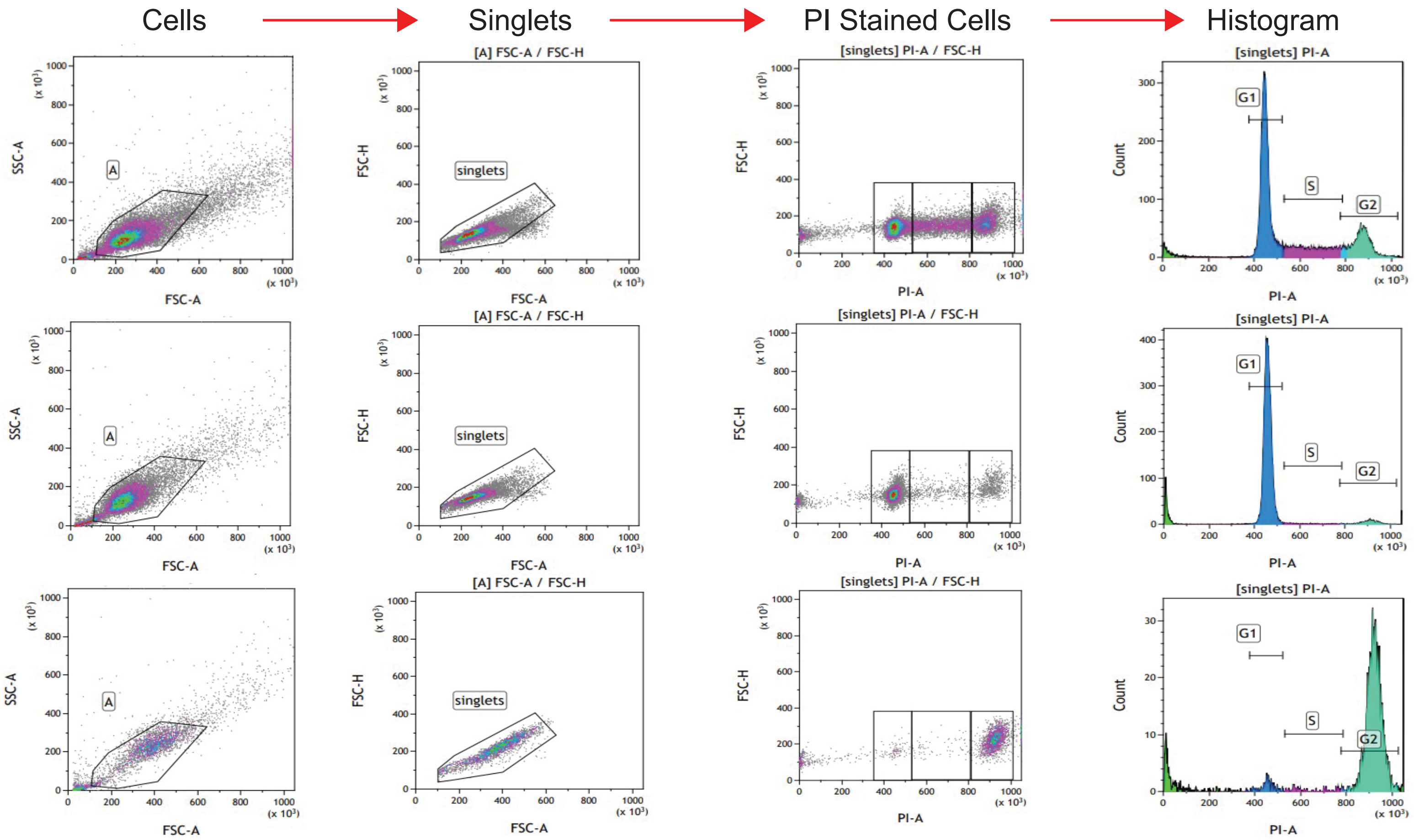

Suppl. Fig. 8 Flow cytometry gating strategy for cell cycle analysis of asynchronous growing, G1 and G2 cells.

Supplemental Figure 9

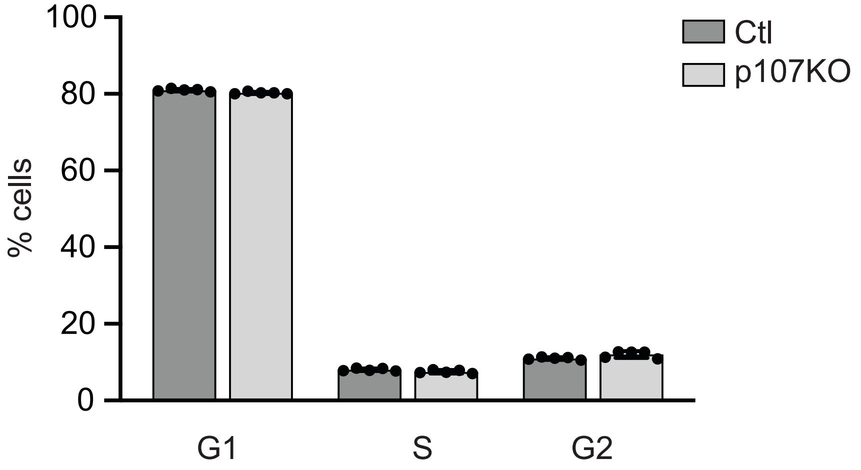

Suppl. Fig. 9 Cell cycle harmonization of control and p107KO c2MP cells. Cell cycle phase profiles of control (Ctl) and p107KO cells that were synchronized by serum starvation to predominately be in the G1 phase of the cell cycle. Data are presented as mean values ± SD (n = 5 biologically independent samples). Data analyzed by two tailed unpaired Student T-test.

Supplemental Figure 10

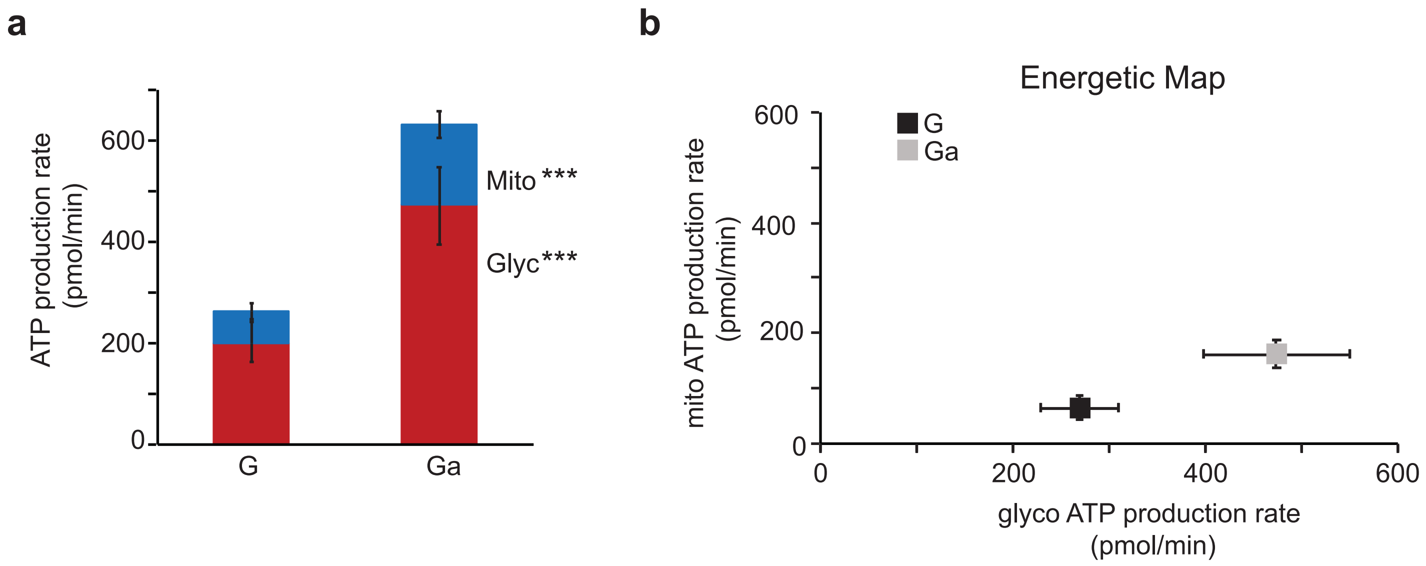

Suppl. Fig. 10 Growth arrested cells have significantly enhanced production of total and mitochondrial ATP. Live cell metabolic analysis by Seahorse of **a** ATP production rate from mitochondria (Mito) and glycolysis (Glyc) and **b** energetic map for proliferating (G) and growth arrested (Ga) c2MPs. Data are presented as mean values ± SD (n = 8 G and 10 Ga biologically independent samples). Two tailed unpaired Student T-test, ***$p<0.001$.

Supplemental Figure 11

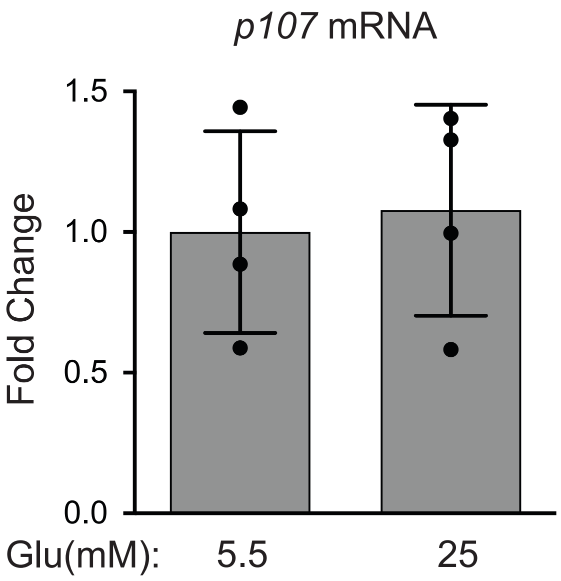

Suppl. Fig. 11 p107 gene expression is unaltered with changes in glucose concentration in stripped media. qPCR analysis for c2MPs grown in stripped media containing only 5.5mM or 25mM glucose (Glu). Data are presented as mean values ± SD (n = 4 biologically independent samples). Data analyzed by two tailed unpaired Student T-test.

Supplemental Figure 12

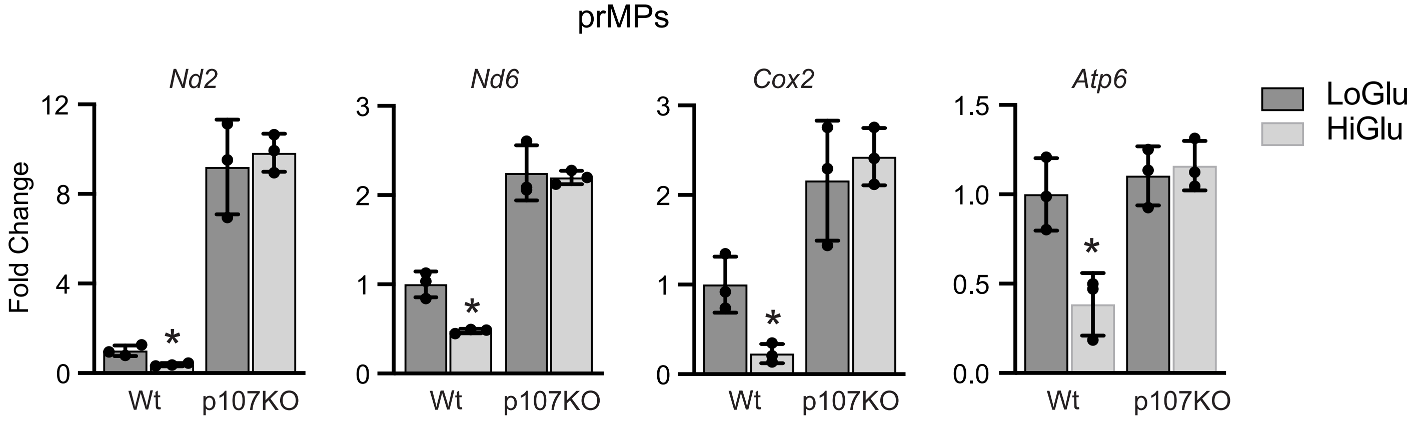

Suppl. Fig. 12 Mitochondrial encoded gene expression analysis of primary myogenic progenitors (prMPs) grown in media with low or high glucose concentrations. Gene expression analysis by RT-qPCR of mitochondrial encoded genes *Nd2*, *Nd6*, *Cox2* and *Atp6* from wild type (Wt) and p107 genetically deleted (p107KO) primary myogenic progenitors (prMPs) grown in stripped media containing 5.5mM (LoGlu) or 25mM (HiGlu) glucose. Data are presented as mean values ± SD (n = 3 biologically independent samples). Two tailed unpaired Student T-test,*p = 0.0366 (*Nd2*), 0.0222 (*Nd6*), 0.0394 (*Cox2*), 0.0171 (*Atp6*).

Supplemental Figure 13

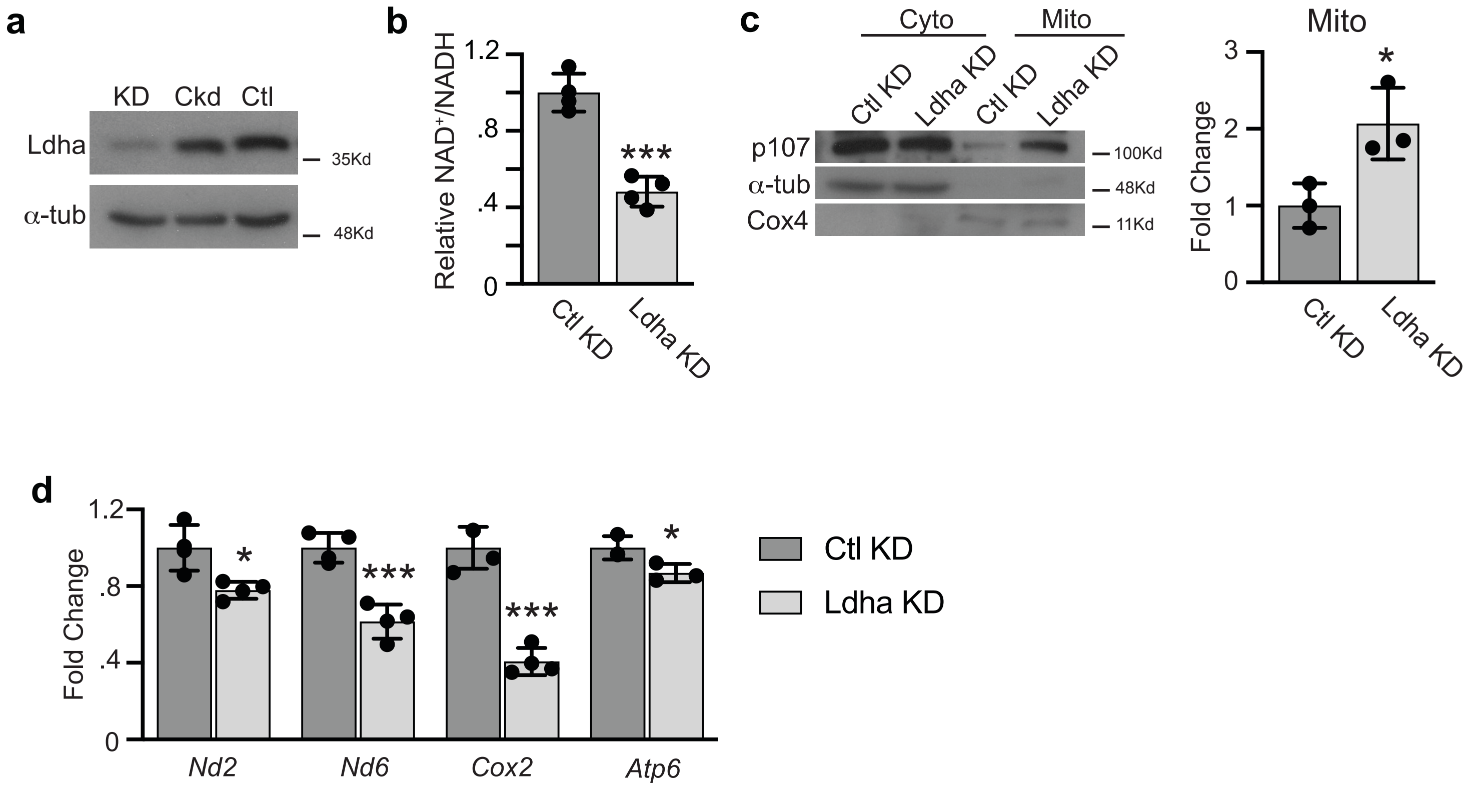

Suppl. Fig. 13 NAD^+/NADH regulation by Ldha knockdown influences p107 mitochondrial function. **a** Representative Western blot for p107 and α-tubulin (α-tub) of c2MPs (Ctl), RNAi knockdown (KD) of Ldha or control knockdown (Ckd) using scrambled RNAi. **b** NAD^+/NADH ratio for control knockdown (Ctl KD) or Ldha KD c2MPs. Data are presented as mean values ± SD (n = 4 biologically independent samples). Two-tailed unpaired Student T-test, ***$p < 0.001$. **c** Representative Western blot and graphical representation of cytoplasmic (Cyto) and mitochondrial (Mito) fractions for p107, α-tubulin (cytoplasmic loading control) and Cox4 (mitochondria loading control) of Ctl KD or Ldha KD. Data are presented as mean values ± SD (n = 3 biologically independent samples). Two-tailed unpaired Student T-test, *$p = 0.037$. **d** Gene expression analysis by RT-qPCR of mitochondrial encoded genes *Nd2, Nd6, Cox2* and *Atp6* for Ctl KD and Ldha KD. Data are presented as mean values ± SD (n = 4 biologically independent samples). Two-tailed unpaired Student T-test, *$p = 0.027$ *Nd2,* 0.0434 *Atp6,* ***$p < 0.001$.

Supplemental Figure 14

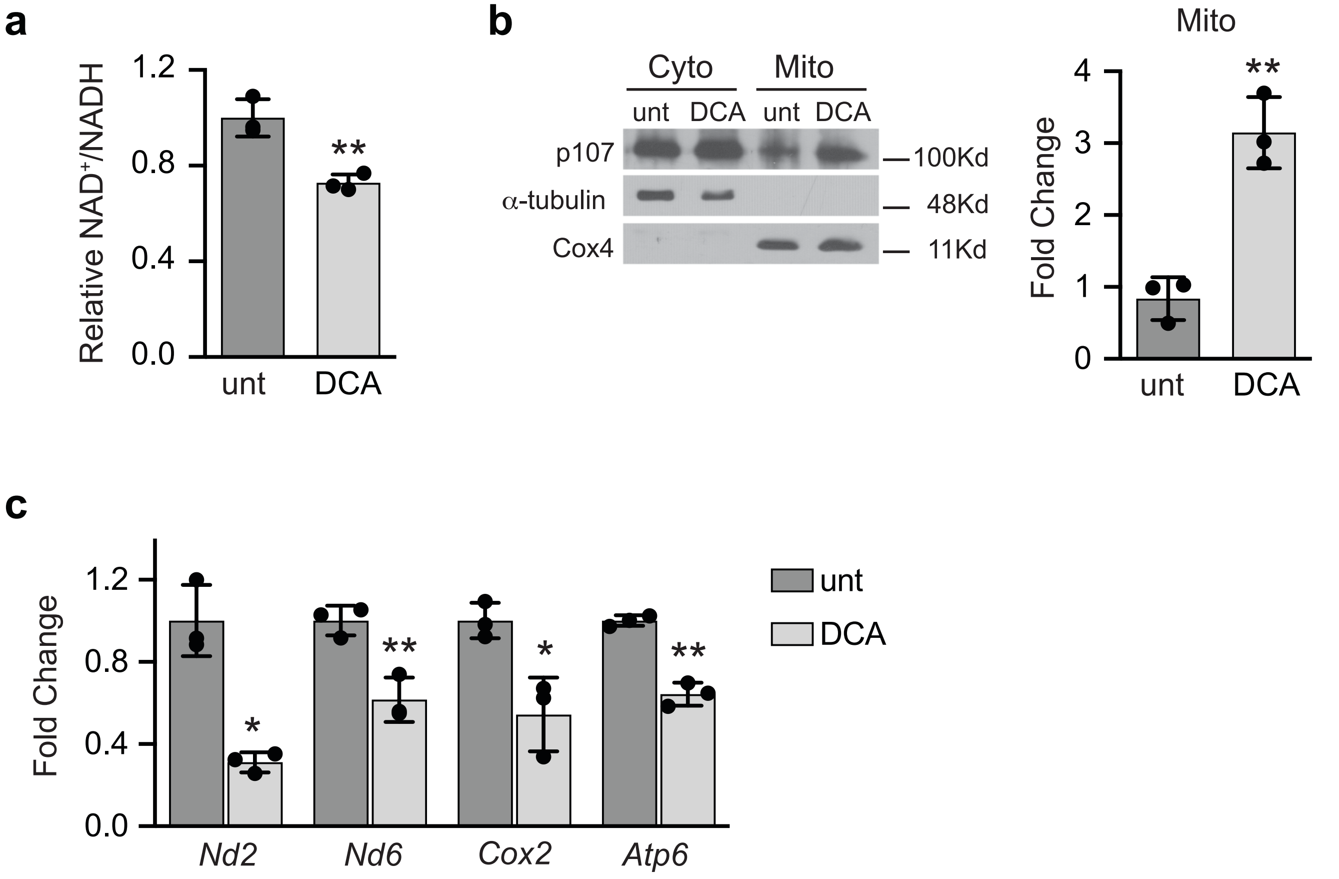

Suppl. Fig. 14 NAD$^+$/NADH regulation by dichloroacetic acid (DCA) influences p107 mitochondrial function. a NAD$^+$/NADH ratio for c2MPs untreated (unt) or treated with dichloroacetic acid (DCA). Data are presented as mean values ± SD (n = 3 biologically independent samples). Two-tailed unpaired Student T-test, $^{**}p = 0.0053$. **b** Representative Western blot and graphical representation of cytoplasmic (Cyto) and mitochondrial (Mito) fractions for p107, α-tubulin (cytoplasmic loading control) and Cox4 (mitochondria loading control) of cells in a. Data are presented as mean values ± SD (n = 3 biologically independent samples). Two-tailed unpaired Student T-test, $^{**}p = 0.0023$. **c** qPCR analysis of cells in a for mitochondrial encoded genes *Nd2*, *Nd6*, *Cox2* and *Atp6*. Data are presented as mean values ± SD (n = 3 biologically independent samples). Two-tailed unpaired Student T-test, $^{*}p = 0.015$ (*Nd2*), $^{*}p = 0.0313$ (*Cox2*), $^{**}p = 0.0096$ (*Nd6*), $^{**}p = 0.0028$ (*Atp6*).

Supplemental Figure 15

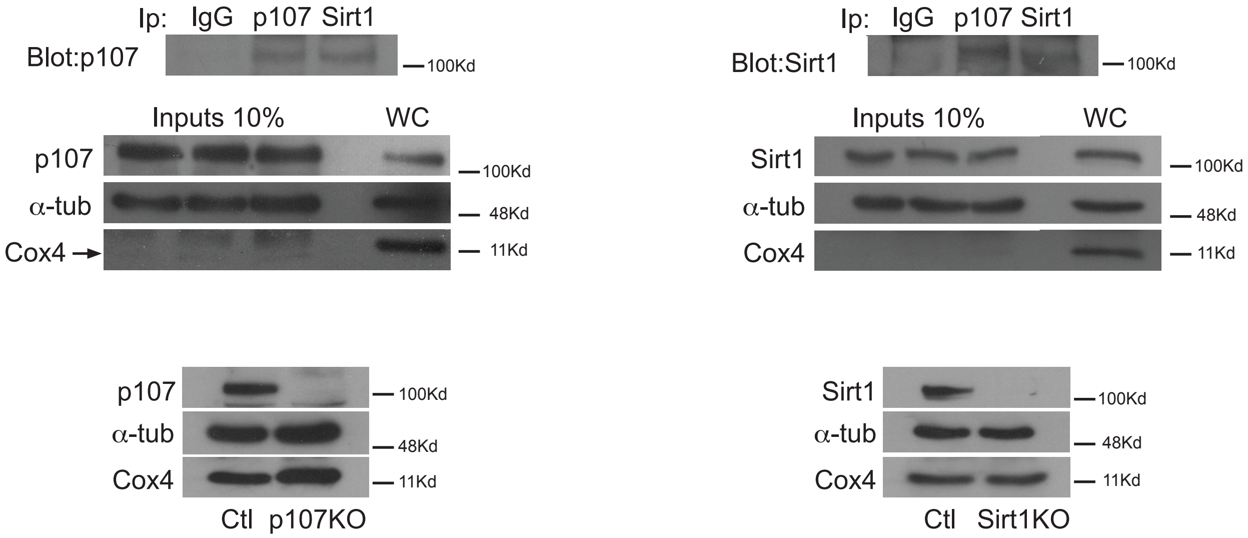

Suppl. Fig. 15 p107 interacts with Sirt1 in cytoplasmic lysates. Immunoprecipitation/Western blots for p107 and Sirt1 from cytoplasmic lysates of c2MP cells. Also Westen blots of inputs and control, p107KO and Sirt1KO c2MPs cells for p107, Sirt1, α-tubulin (α-tub) and Cox4.

Supplemental Figure 16

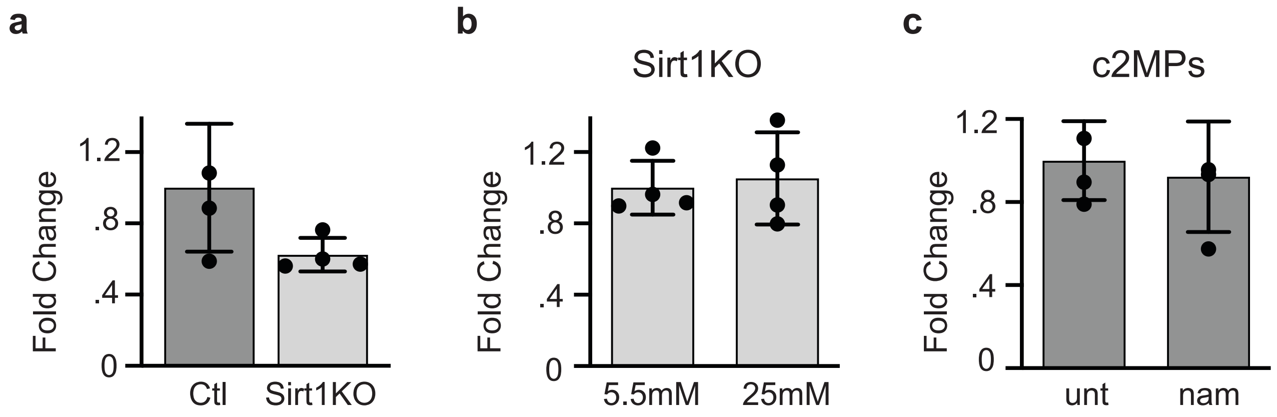

Suppl. Fig. 16 p107 gene expression is unaltered in Sirt1KO cells and nicotinamide treated c2MP cells. RT-qPCR analysis for p107 from **a** control (Ctl) and Sirt1KO c2MPs grown in 5.5mM glucose. Data are presented as mean values ± SD (n = 4 biologically independent samples). **b** Sirt1KO c2MPs grown in stripped media containing only 5.5mM or 25mM glucose. Data are presented as mean values ± SD (n = 4 biologically independent samples). **c** untreated (unt) or nicotinamide treated c2MPs. Data are presented as mean values ± SD (n = 3 biologically independent samples). Data analyzed by two tailed unpaired Student T-test.

Supplemental Figure 17

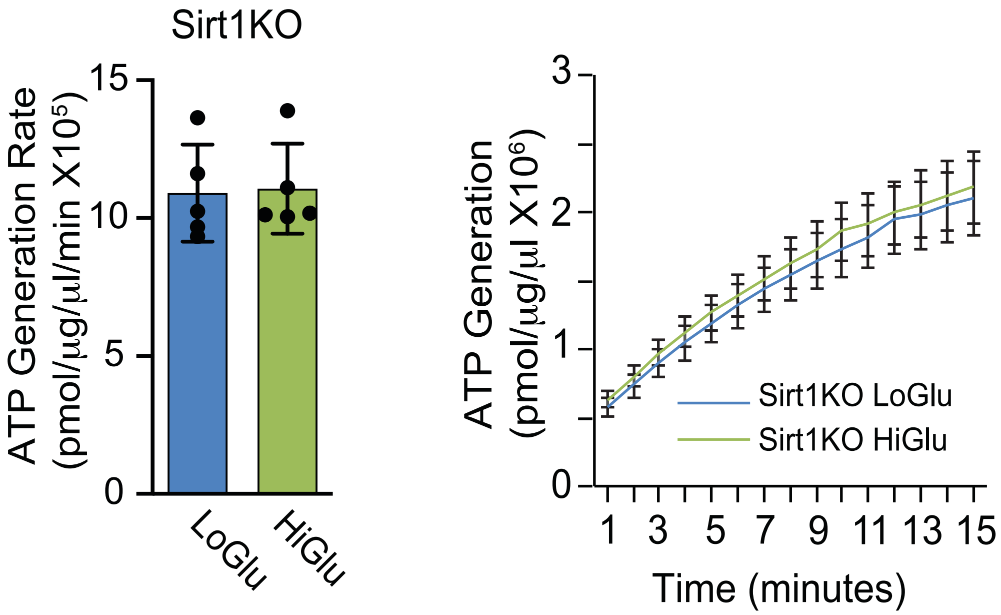

Suppl. Fig. 17 Varying the glucose concentrations does not influence ATP generation rate and capacity in isolated mitochondria of Sirt1KO cells. Isolated mitochondrial ATP generation rate and capacity over time for Sirt1KO c2MPs grown in 5.5mM (LoGlu) and 25mM (HiGlu) glucose. Data are presented as mean values ± SD (n = 5 biologically independent samples). Data analyzed by two tailed unpaired Student T-test.

Supplemental Figure 18

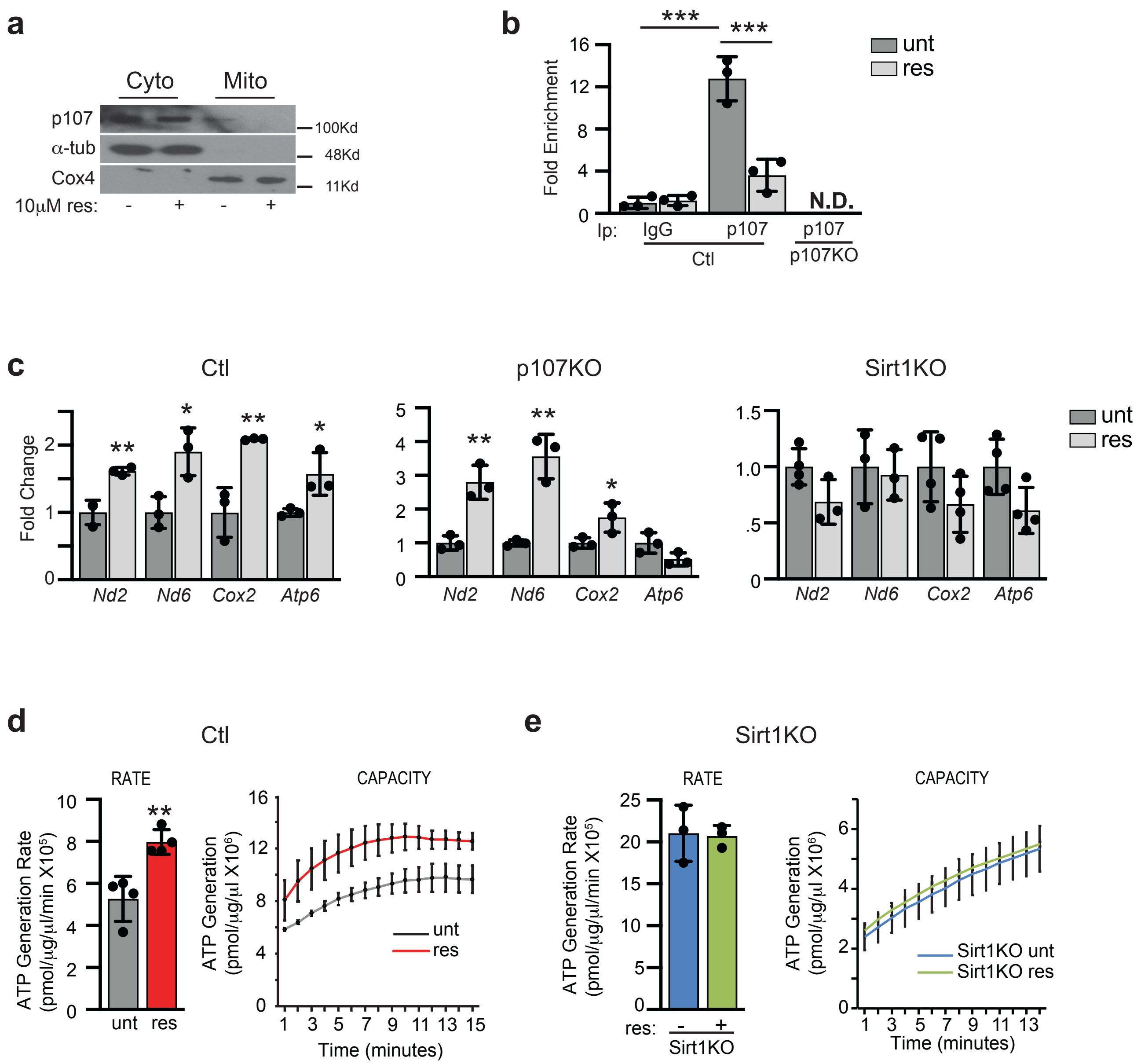

Suppl. Fig. 18 A low resveratrol (10μM) concentration that activates Sirt1 decreases p107 mitochondrial function. a Representative Western blot of Cyto and Mito fractions of c2MPs untreated (Ctl) or treated with a concentration (10μM) of resveratrol (res) that activates Sirt1. **b** Graphical representation of relative p107 and IgG interaction at mitochondrial DNA by qChIP analysis in Ctl and p107KO c2MPs untreated (unt) or treated with a concentration of res (10μM) that activates Sirt1. Data are presented as mean values ± SD (n = 3 biologically independent samples). Two-way Anova with post hoc Tukey, ***$p<0.001$. **c** RT-qPCR of *Nd2, Nd6, Cox2* and *Atp6* for Ctl, p107KO and Sirt1KO c2MPs unt and treated for 10μM res. Data are presented as mean values ± SD (n = 3 Ctl, p107KO and 4 Sirt1KO biologically independent samples). Two-tailed unpaired Student T-test; Ctl: *p = 0.0211 (*Nd6*), 0.0364 (*Atp6*), **p = 0.0049 (*Nd2*), 0.003 (*Cox2*), p107KO: *p = 0.0488 (*Cox2*), **p = 0.0047 (*Nd2*), 0.0026 (*Nd6*). Isolated mitochondria ATP generation rate and capacity over time for **d** Ctl (n = 4 biologically independent samples) and **e** Sirt1KO c2MPs (n = 4 biologically independent samples) treated and untreated with with 10μM res. Data are presented as mean values ± SD. Two tailed unpaired Student T-test, **p = 0.0081. For capacity significance statistics see Supplementary Table 1f.

Supplemental Figure 19

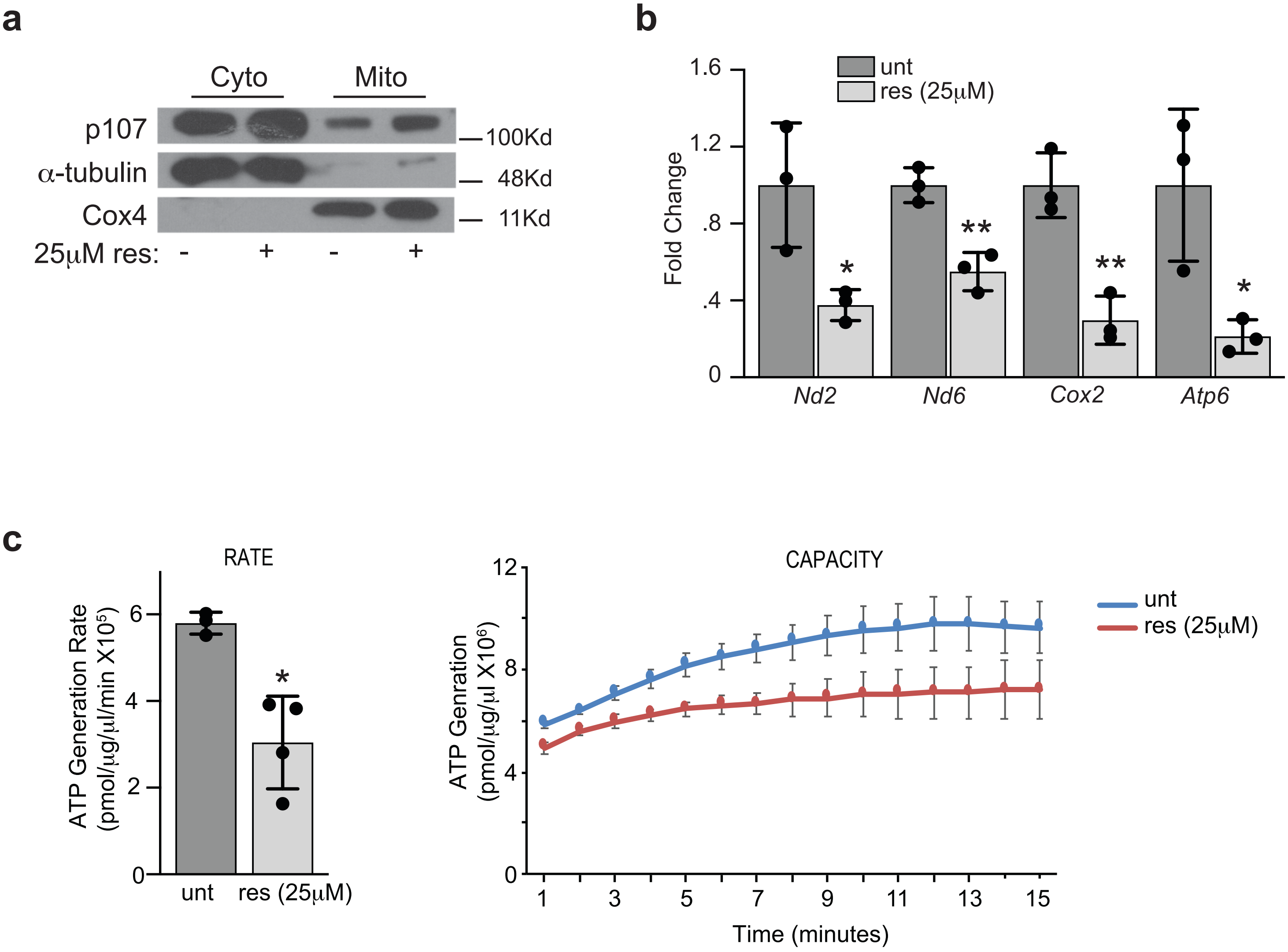

Suppl. Fig. 19 A high resveratrol (25µM) concentration that inactivates Sirt1 increases p107 mitochondrial function. a Representative Western blot of cytoplasmic (Cyto) and mitochondrial (Mito) fractions for p107, α-tubulin and Cox4 of c2MPs untreated (unt) or treated with a concentration (25µM) of resveratrol (res) that inactivates Sirt1. **b** Gene expression analysis by RT-qPCR of mitochondrial encoded genes *Nd2*, *Nd6*, *Cox2* and *Atp6* for c2MPs untreated (unt) or treated with a concentration (25µM) of res that inactivates Sirt1. Data are presented as mean values ± SD (n = 3 biologically independent samples). Two-tailed unpaired Student T-test, *p = 0.0318 (*Nd2*), 0.0301 (*Atp6*), **p = 0.0044 (*Cox2*), p = 0.0049 (*Nd6*). **c** Isolated mitochondrial ATP generation rate and capacity over time for c2MPs untreated (unt) or treated with a concentration (25µM) of res that inactivates Sirt1. Data are presented as mean values ± SD (n = 4 biologically independent samples). Two-tailed unpaired Student T-test, *p = 0.0112. For capacity significance statistics see Supplementary Table 1g.

Supplemental Figure 20

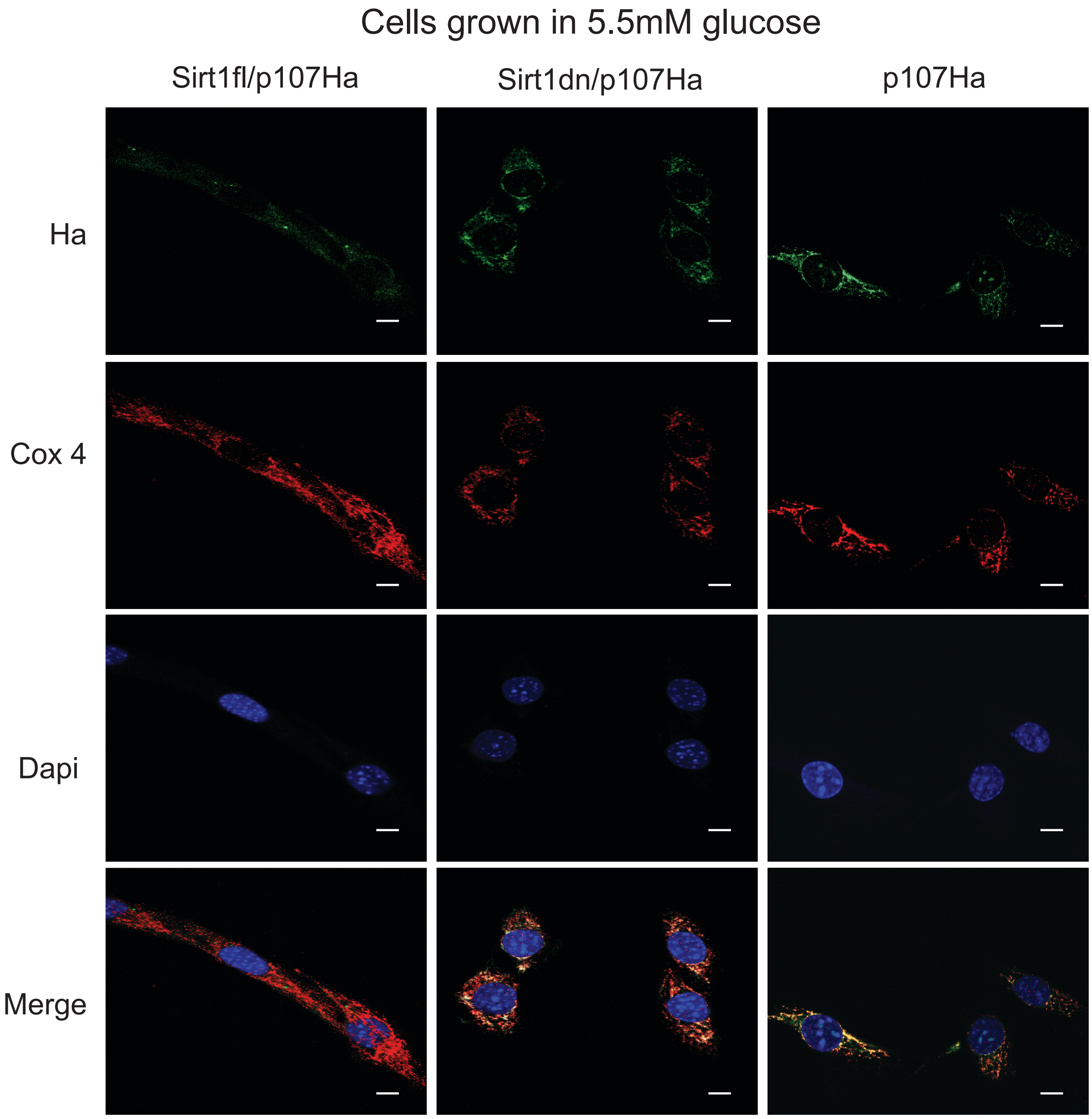

Suppl. Fig. 20 Confocal immunofluorescence microscopy for Ha (green), Cox4 (red), Dapi (blue) and Merge of c2MPs transfected with full length Ha-tagged p107 (p107Ha) alone or with full length Sirt1 (Sirt1-fl) or dominant negative Sirt1 (Sirt1dn) then grown in stripped media containing 5.5mM glucose (scale bar 10μm).

Supplemental Figure 21

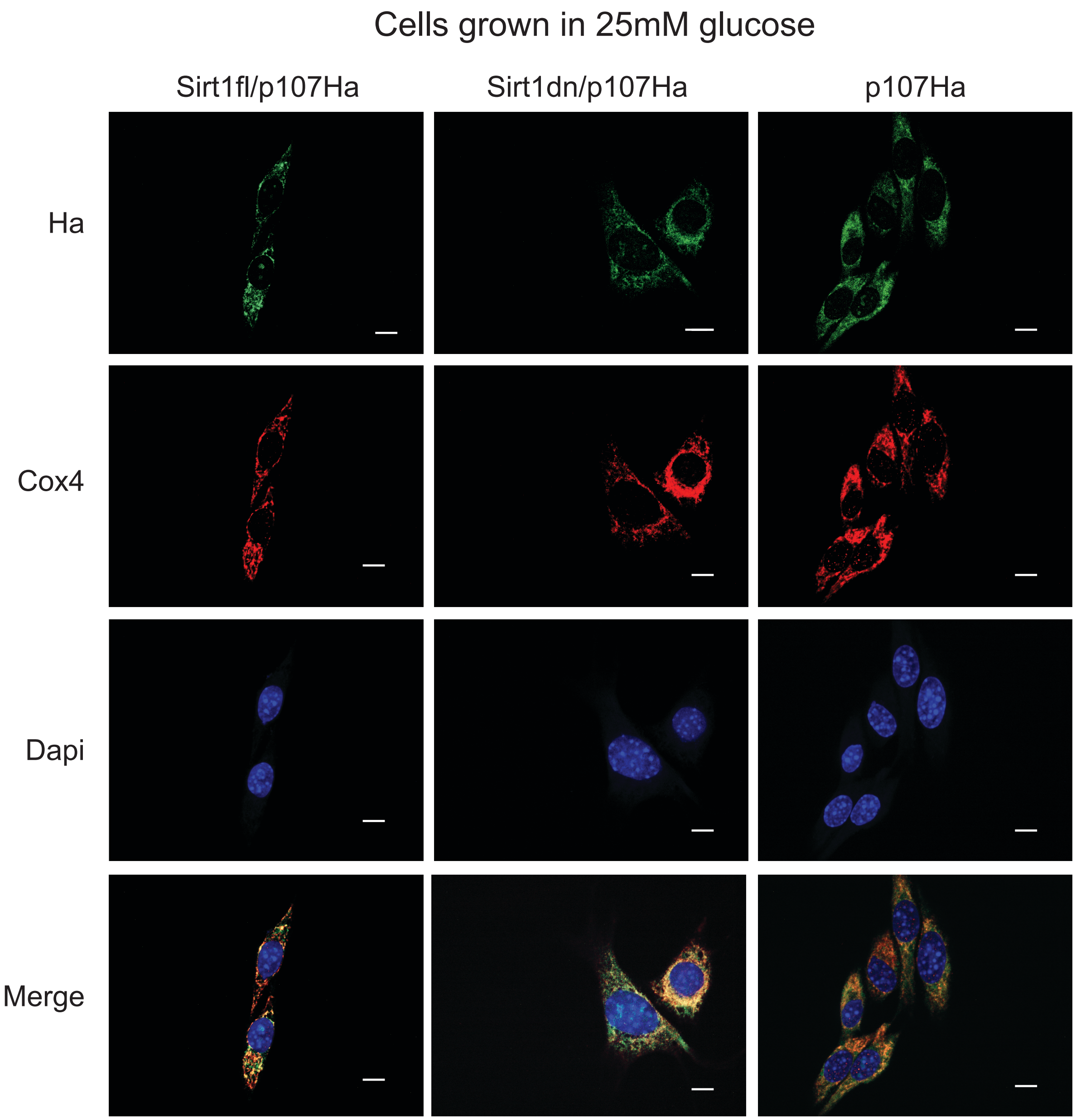

Suppl. Fig. 21 Confocal immunofluorescence microscopy for Ha (green), Cox4 (red), Dapi (blue) and Merge of c2MPs transfected with full length Ha-tagged p107 (p107Ha) alone or with full length Sirt1 (Sirt1-fl) or dominant negative Sirt1 (Sirt1dn) then grown in stripped media containing 25mM glucose (scale bar 10μm).

Supplemental Figure 22

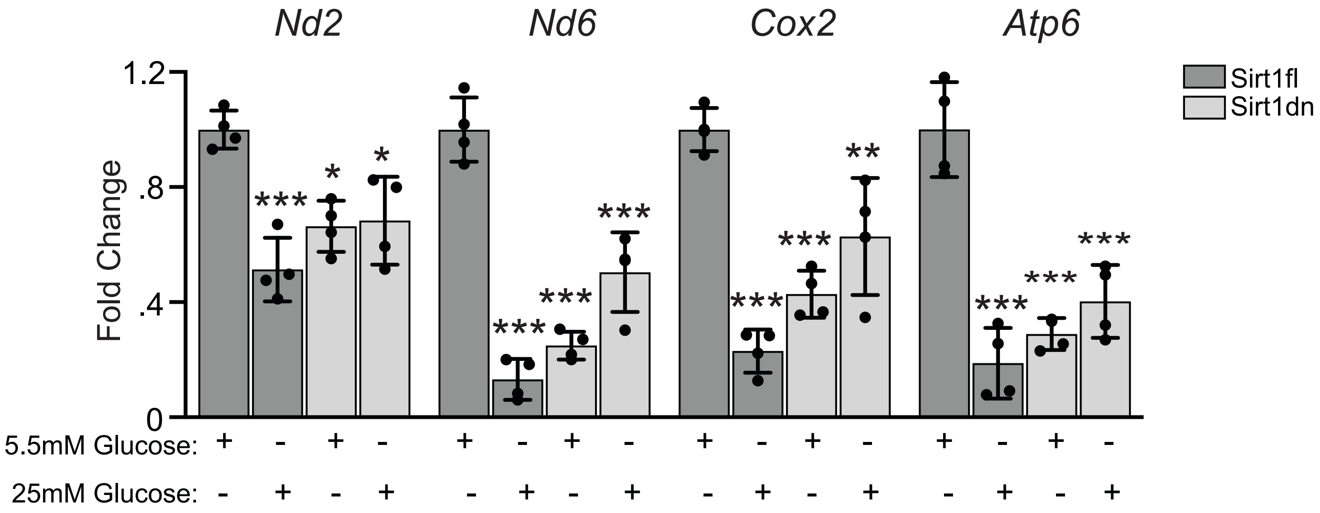

Suppl. Fig. 22 Gene expression analysis by RT-qPCR of mitochondrial encoded genes *Nd2*, *Nd6*, *Cox2* and *Atp6* of c2MPs transfected with full length (Sirt1fl) or dominant negative (Sirt1dn) grown in stripped media containing 5.5mM or 25mM glucose. Data are presented as mean values ± SD (n = 4, biologically independent samples). Two-way Anova with post hoc Tukey, *$p = 0.045$ Sirt1dn 5.5mM, 0.0254 Sirt1dn 25mM, **$p = 0.008$ Sirt1dn 25mM, ***$p < 0.001$.

Supplemental Figure 23

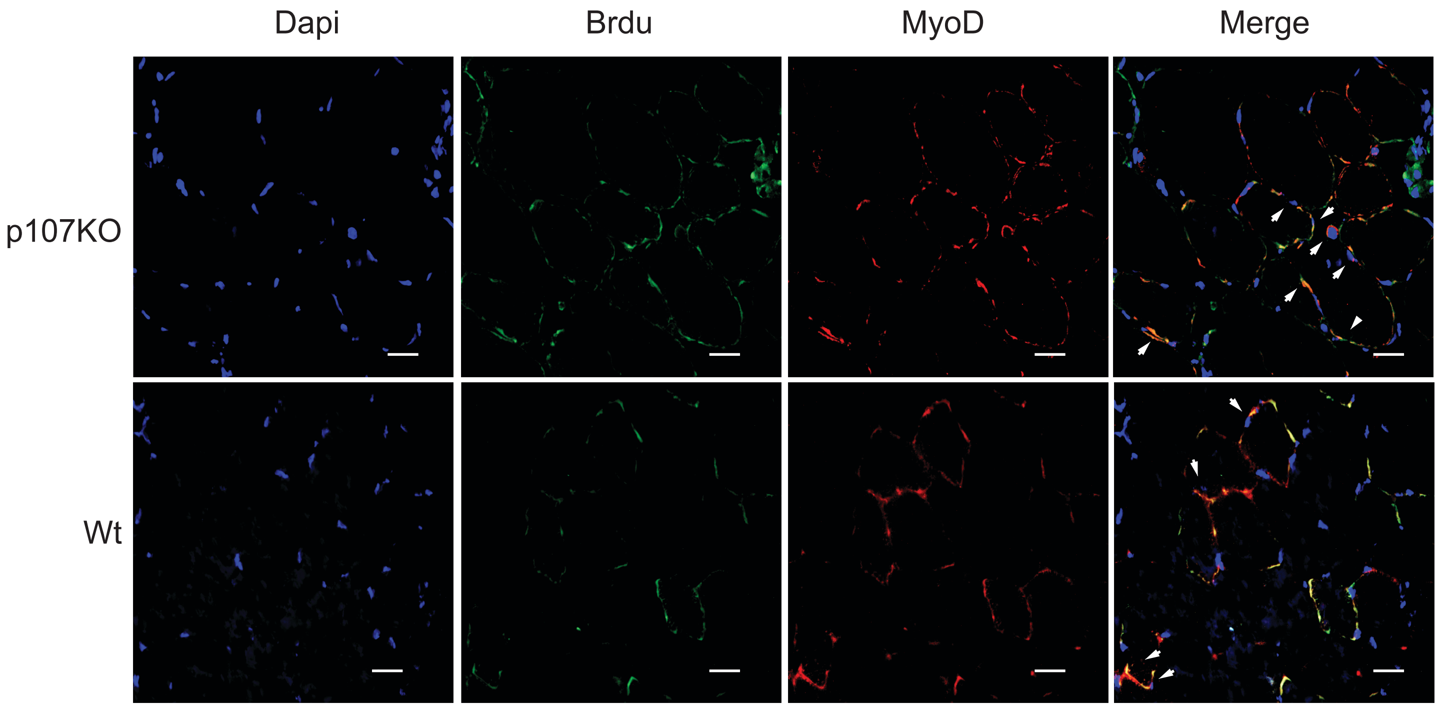

Suppl. Fig. 23 Fluorescent channels corresponding to images in Figure 5a. Confocal immunofluorescence microscopy for Brdu (green), MyoD (red), Dapi (blue) and Merge of tibialis anterior muscle tissue sections from wild type (Wt) and p107 genetically deleted (p107KO) mice 2 days post injection with cardiotoxin that had been treated with bromodeoxyuridine (Brdu) on the previous day (scale bar 20μm). Arrows denote Brdu and MyoD positive nuclei.

Supplemental Figure 24

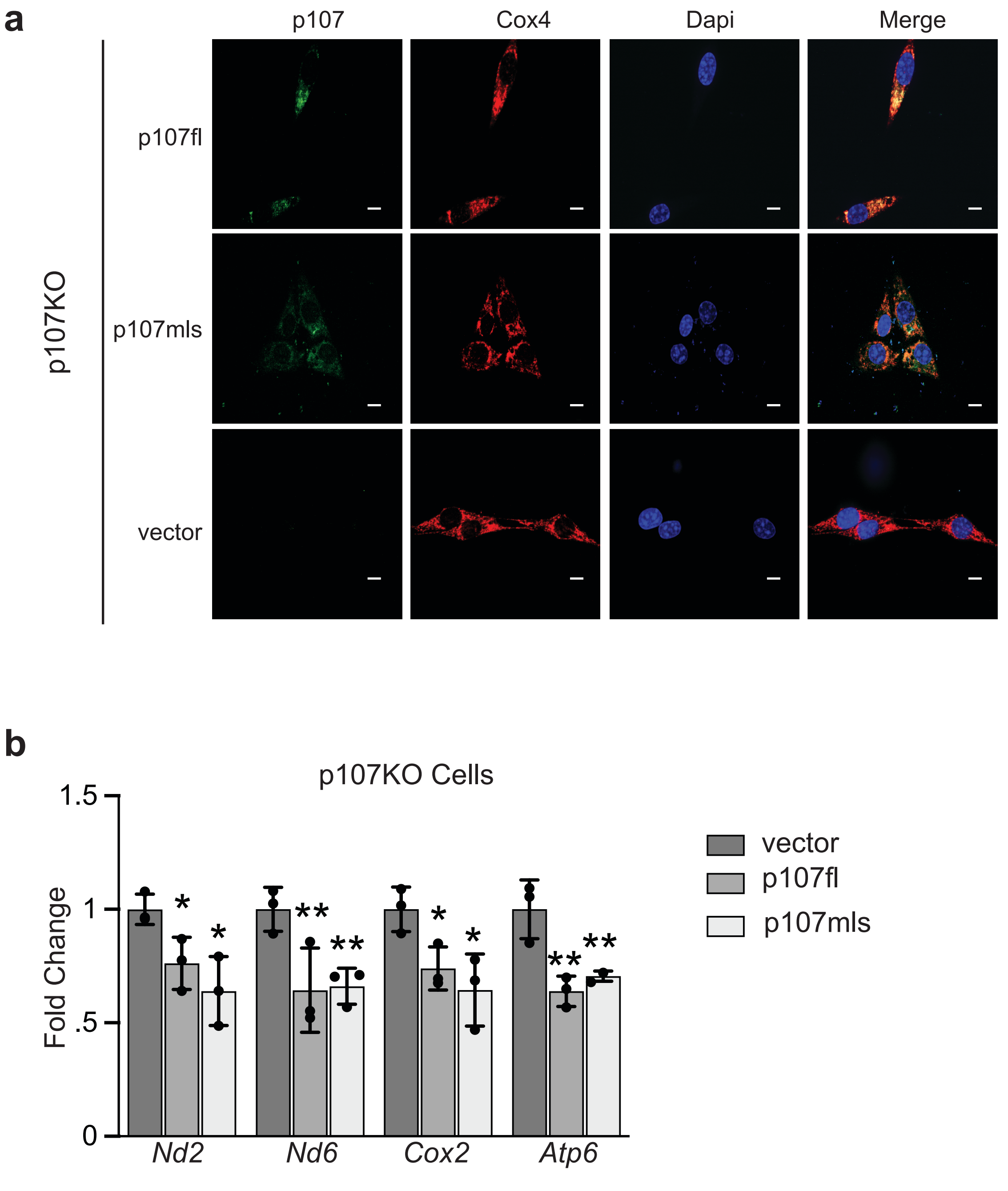

Suppl. Fig. 24 p107 mitochondrial localization in p107KO MPs down-regulates mtDNA gene expression. p107 genetically deleted (p107KO) c2MPs transfected with either empty vector alone or together with full length p107 (p107fl) or mitochondria localized p107 (p107mls) were assessed by **a** confocal immunofluorescence for p107 (green), Cox4 (red), Dapi (blue) and Merge (scale bar 10μm) and **b** Gene expression analysis by RT-qPCR of mitochondrial encoded genes *Nd2*, *Nd6*, *Cox2* and *Atp6*. Data are presented as mean values ± SD (n = 3 biologically independent samples). One way-Anova with post hoc Tukey, *Nd2* $*p$ = 0.0312 (p107fl), 0.0121 (p107mls), *Nd6* $**p$ = 0.01 (p107fl), 0.01 (p107mls), *Cox2* $*p$ = 0.042 (p107fl), 0.0267 (p107mls), *Atp6* $**p$ = 0.0049 (p107fl), 0.009 (p107mls).

Supplemental Figure 25

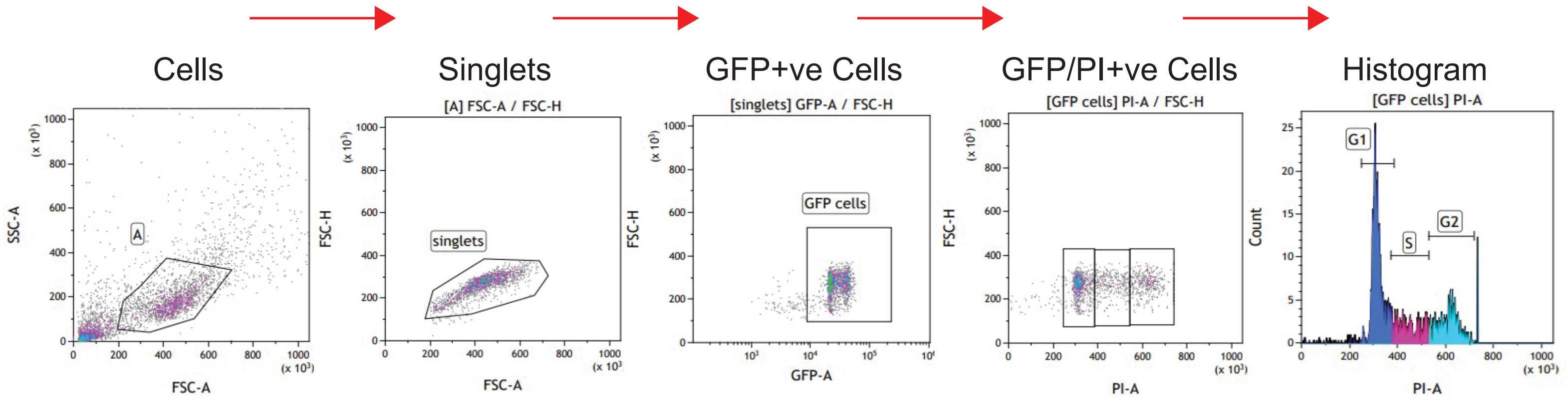

Suppl. Fig. 25 Flow cytometry gating strategy for cell cycle analysis of asynchronous GFP expressing cells.

Supplemental Figure 26

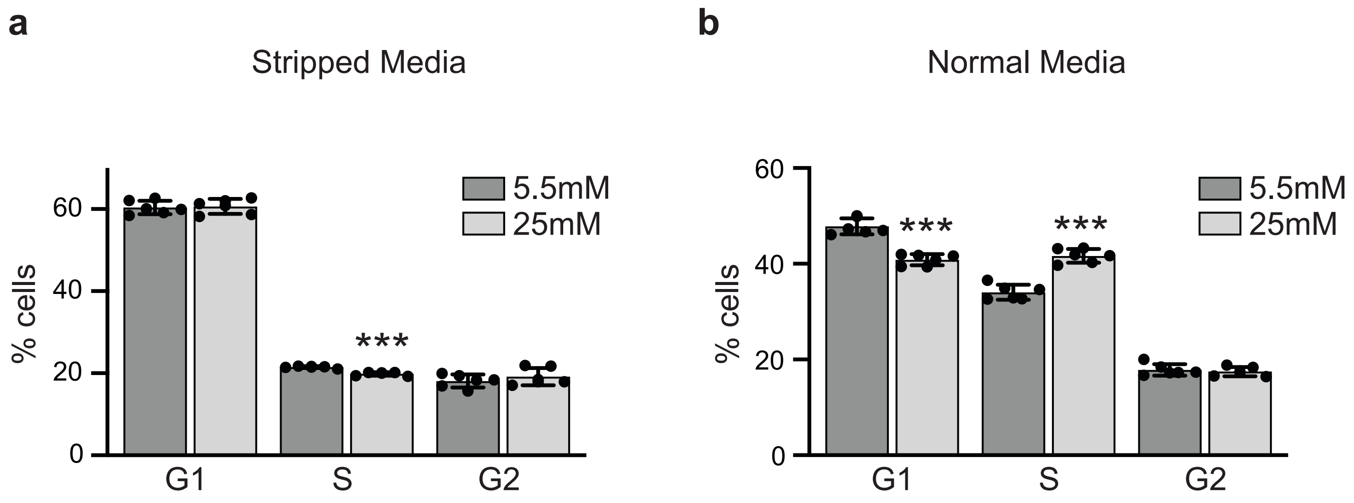

Suppl. Fig. 26 Cell cycle profile for varied glucose concentration in stripped or complete media. G1, S and G2 cell cycle analysis for **a** c2MPs grown in stripped media containing only 5.5mM or 25mM glucose as the sole nutrient and **b** cMPS grown in normal media with 5.5mM or 25mM glucose. Data are presented as mean values ± SD (n = 6 biologically independent samples). Two-tailed unpaired Student T-test, ****p* < 0.001.

Supplemental Figure 27

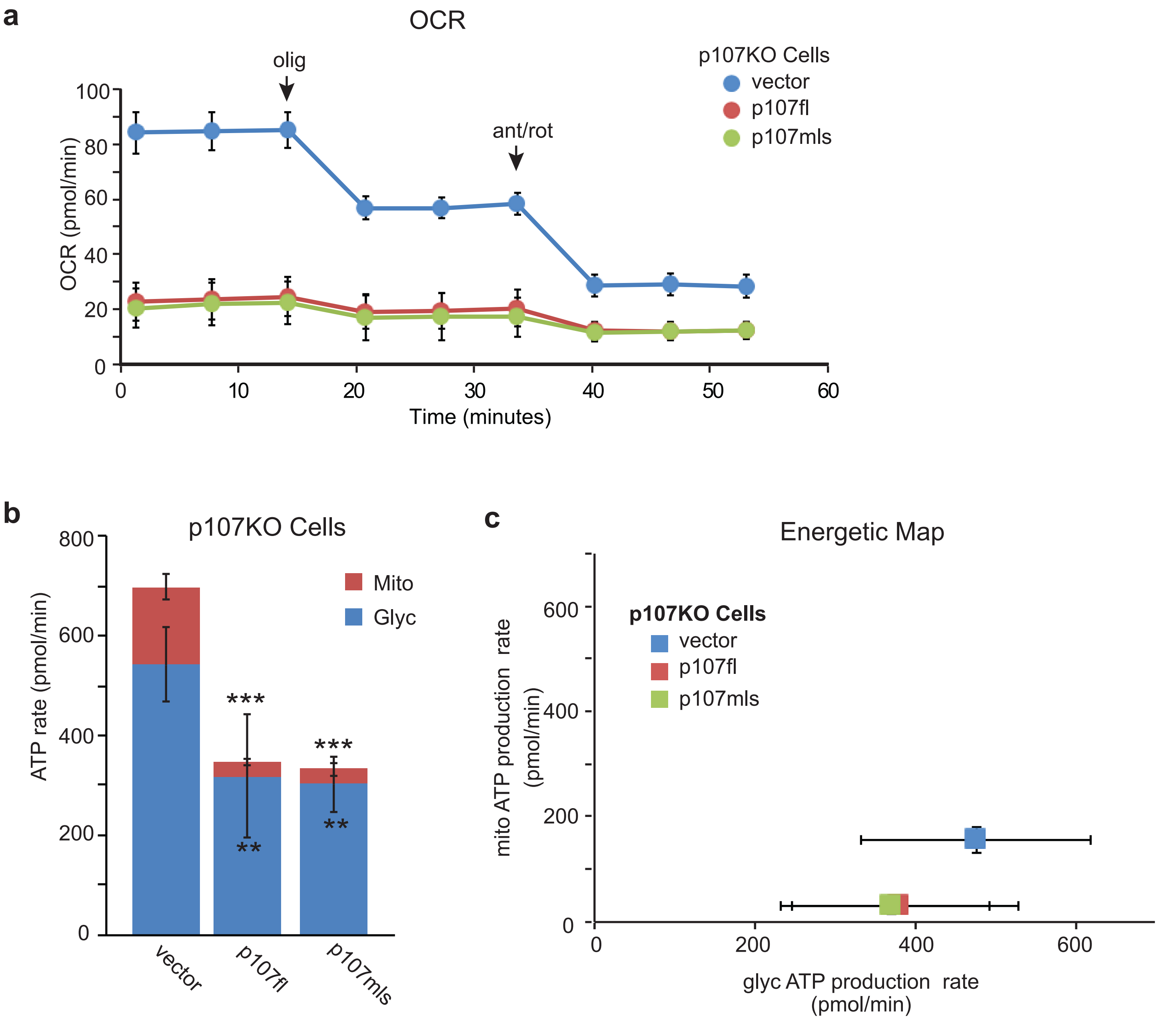

Suppl. Fig. 27 p107 mitochondrial localization in p107KO MPs reduces production of total and mitochondrial ATP. Live cell metabolic analysis by Seahorse of **a** Oxygen consumption rate (OCR) with addition of oligomycin (olig) and antimycin A (ant)/rotenone (rot), **b** ATP production rate from mitochondria (Mito) and glycolysis (Glyc) and **c** energetic map for proliferating p107 genetically deleted (p107KO) c2MPs transfected with either empty vector or together with full length p107 (p107fl) or mitochondria localized p107 (p107mls). Data are presented as mean values ± SD (n = 12 vector, 6 p107fl and 6 p107mls biologically independent samples). One-way Anova with post hoc Tukey, for Glyc $**p = 0.0049$ (p107fl), 0.0031 (p107mls), $***p < 0.001$.

Supplemental Figure 28

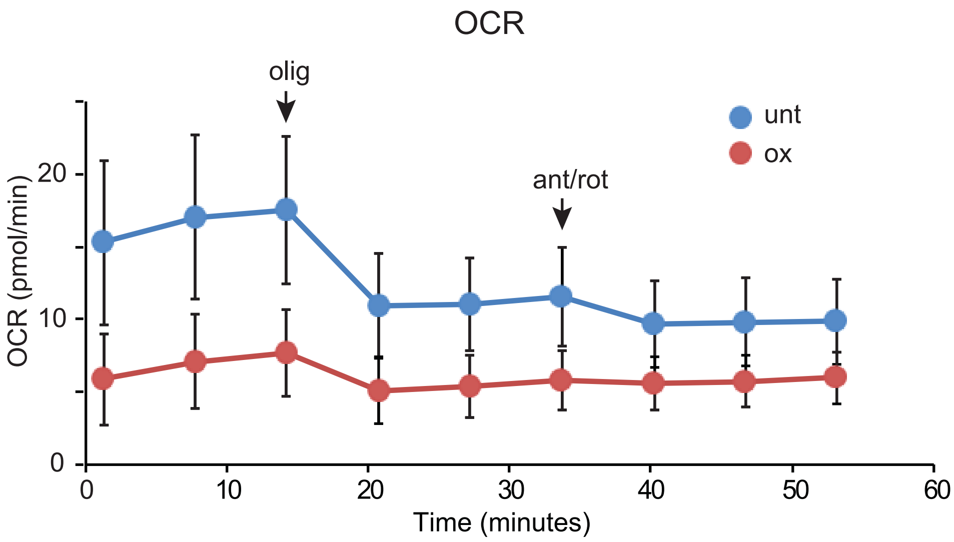

Suppl. Fig. 28 Oxamate treated cells have reduced oxygen consumption rate (OCR). Live cell metabolic analysis by Seahorse of Oxygen consumption rate (OCR) with addition of oligomycin (olig) and antimycin A (ant)/rotenone (rot) for c2MPs untreated (unt) or treated oxamate (ox). Data are presented as mean values ± SD (n = 8 unt and 11 ox biologically independent samples).

Supplemental Figure 29

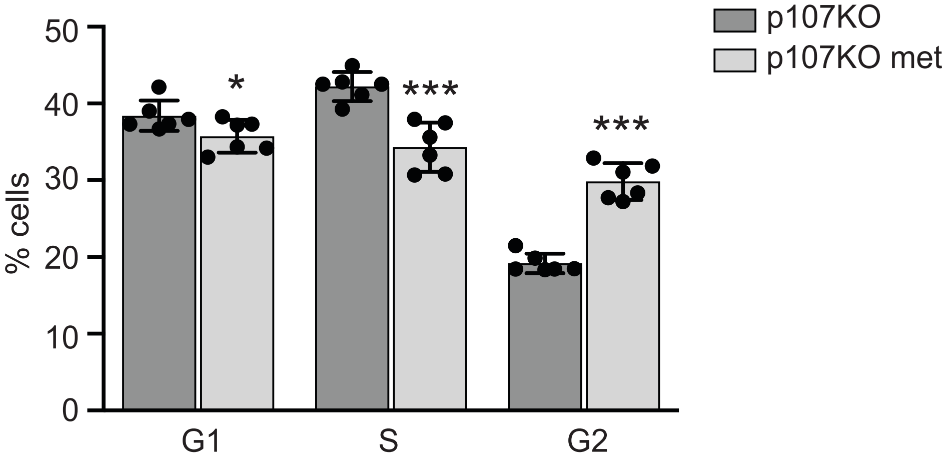

Suppl. Fig. 29 G1, G2 and S cell cycle phases are reduced in p107KO cells with metformin treatment. Cell cycle analysis by flow cytometry for p107KO c2MPs untreated or treated with metformin (met). Data are presented as mean values ± SD (n = 6 biologically independent samples). Two-tailed unpaired Student T-test, *p = 0.0475, ***p<0.001.

Supplemental Figure 30

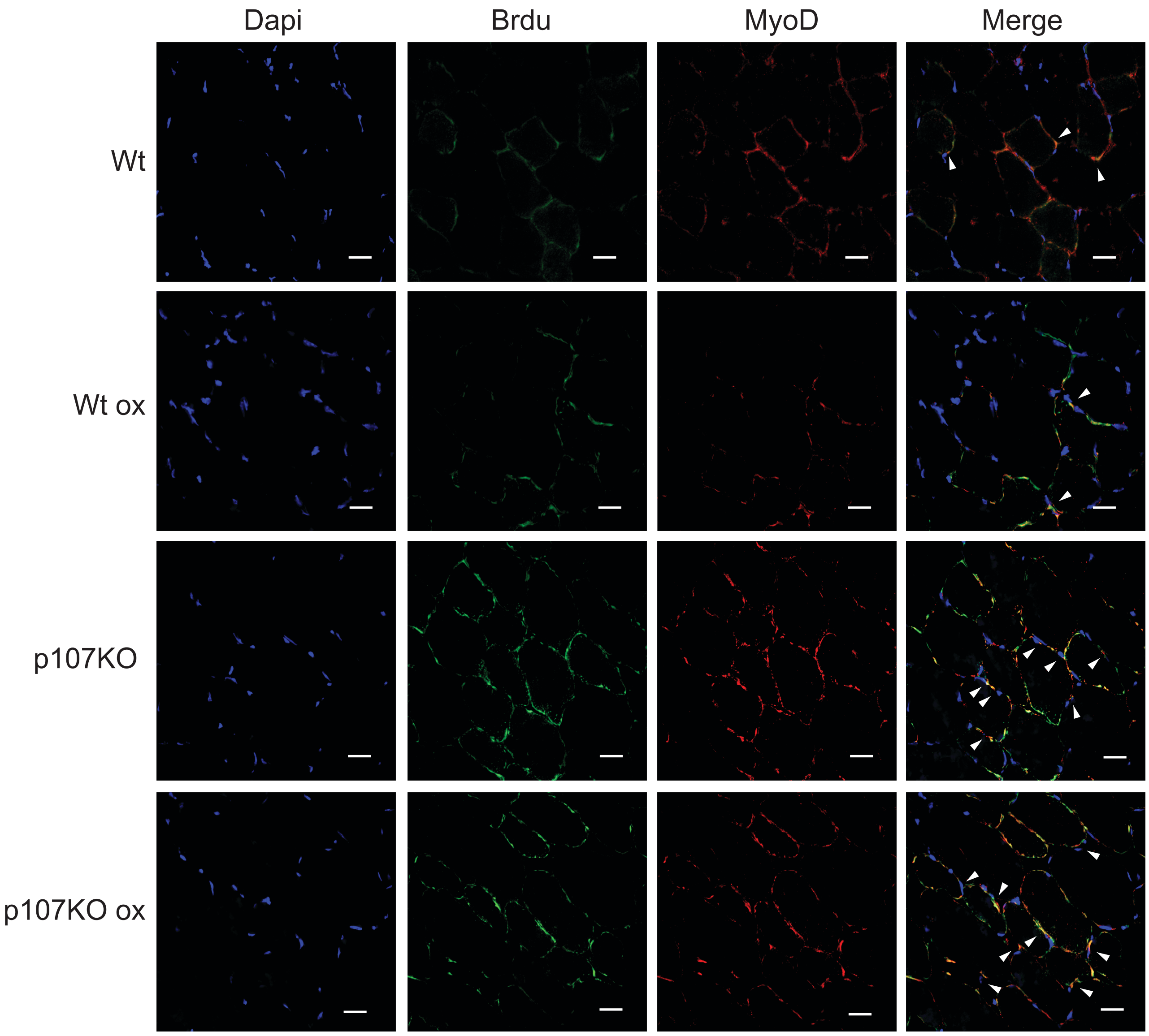

Suppl. Fig. 30 Fluorescent channels corresponding to images in Figure 5o. Confocal immunofluorescence microscopy for Brdu (green), MyoD (red), Dapi (blue) and Merge of tibialis anterior muscle tissue section from wild type (Wt) and p107 genetically deleted (p107KO) mice 2 days post injection with cardiotoxin that had been treated with bromodeoxyuridine (Brdu) on the previous day in the absence or presence of oxamate (ox) for 4 days. Arrows denote Brdu and MyoD positive nuclei (scale bar 20μm).

Supplemental Table 1. Statistical significance values at each timepoint for various ATP generation capacity graphs.

**a ATP generation capacity for proliferation (G) and growth arrest (Ga), n=4 (Fig. 2i)**	
**Time (min.)**	**Significance**
1	0.033131343 (*)
2	0.016586179 (*)
3	0.011167771 (*)
4	0.00665779 (**)
5	0.005020464 (**)
6	0.005420062 (**)
7	0.001023739 (***)
8	0.002013576 (**)
9	0.001840361 (**)
10	0.001772278 (**)
11	0.001374734 (**)
12	0.001369558 (**)
13	0.001820507 (**)
14	0.000942071 (***)
15	0.000710462 (***)

**b ATP generation capacity for proliferating control (Ctl) and p107KO c2MPs, n=4 (Fig. 2j)**	
**Time (min.)**	**Significance**
1	0.009417287 (**)
2	0.008062857 (**)
3	0.005542089 (**)
4	0.00526662 (**)
5	0.004568636 (**)
6	0.008806086 (**)
7	0.015920646 (*)
8	0.04541628 (*)
9	0.018155165 (*)
10	0.020427915 (*)
11	0.0183923 (*)
12	0.020811991 (*)
13	0.021514531 (*)
14	0.021540274 (*)
15	0.003472532 (**)

c ATP generation capacity for c2MPs grown in SM containing 5.5mM and 25mM glucose, n=4, (Fig. 3g)	
**Time (min.)**	**Significance**
1	0.035331802 (*)
2	0.030200259 (*)
3	0.025596033 (*)
4	0.024064309 (*)
5	0.02435088 (*)
6	0.022419532 (*)
7	0.021898214 (*)
8	0.020053804 (*)
9	0.017606462 (*)
10	0.009207275 (**)
11	0.011190924 (*)
12	0.036293605 (*)
13	0.016513239 (*)
14	0.011994536 (*)
15	0.005802656 9 (**)

d ATP generation capacity for Ctl and Sirt1KO treated with 5.5mM glucose, n=4 (Fig. 4d)	
**Time (min.)**	**Significance**
1	0.001807382 (**)
2	0.000423724 (***)
3	0.004639819 (**)
4	0.000760897 (***)
5	0.001120419 (**)
6	0.000853747 (***)
7	0.000975817 (***)
8	0.001268889 (**)
9	0.000954513 (***)
10	0.001572121 (**)
11	0.001643781 (**)
12	0.001953617 (**)
13	0.001685461 (**)
14	0.002527076 (**)
15	0.002392154 (**)

e ATP generation capacity for c2MPs treated or untreated with 10mM nam, n=4 (Fig. 4h)	
**Time (min.)**	**Significance**
1	0.000360044 (***)
2	0.006963349 (**)
3	0.014770086 (*)
4	0.02132306 (*)
5	0.022632029 (*)
6	0.032207431 (*)
7	0.034483084 (*)
8	0.035670347 (*)
9	0.007821973 (**)
10	0.020493916 (*)
11	0.027989954 (*)
12	0.032556922 (*)
13	0.038031967 (*)
14	0.034633286 (*)
15	0.032465285 (*)

f ATP generation capacity for c2MPs treated or untreated with 10µM res, n=4 (Suppl. Fig. 18d)	
**Time (min.)**	**Significance**
1	0.064444463 (*)
2	0.027493958 (*)
3	0.021748346 (*)
4	0.016031546 (*)
5	0.01277941 (*)
6	0.010063891 (**)
7	0.006263203 (**)
8	0.0040332 (**)
9	0.002687763 (**)
10	0.00280281 (**)
11	0.00231082 (**)
12	0.005146623 (**)
13	0.005146623 (**)
14	0.00451611 (**)
15	0.005339423 (**)

g ATP generation capacity for c2MPs treated or untreated with 25µM res, n=4 (Suppl. Fig. 19c)	
**Time (min.)**	**Significance**
1	0.000959268 (***)
2	0.001423853 (**)
3	0.00096012 (***)
4	0.000862286 (***)
5	0.001400103 (**)
6	0.001343943 (**)
7	0.001396845 (**)
8	0.0025641 (**)
9	0.003482068 (**)
10	0.006695159 (**)
11	0.008173165 (**)
12	0.010742694 (*)
13	0.010742694 (**)
14	0.017190247 (*)
15	0.019641938 (*)

Supplemental Table 2

Figure 2n statistical significance values of mitochondria number per grouped length (two-tailed unpaired Student T-test)

Length Range (μm)	Significance
0.2 to 0.4	0.0104 (**)
0.41 t0 0.6	0.0042 (**)
0.61 to 0.8	0.0023 (**)
0.81 to 1	0.0084 (**)
1.01 to 1.2	0.7907 (ns)
1.21 to 1.4	0.0027 (**)
1.41 to 1.6	0.0012 (**)
1.61 to 1.8	< 0.001 (***)
1.81 to 2	< 0.001 (***)
2.01 to 2.2	0.0012 (**)
2.21 to 2.4	< 0.001 (***)
above 2.4	< 0.001 (***)

Figure 2n statistical significance values of mitochondria number per grouped area (two-tailed unpaired Student T-test)

Area Range (μm^2)	Significance
.01 to.04	0.0626 (ns)
0.041 to .08	0.5337 (ns)
.081 to .12	0.5093 (ns)
.121 to .16	0.0383 (*)
.161 to .2	0.0222 (*)
.21 to .6	0.0020 (**)
.61 to 1	0.0012 (**)
1.1 to 2	< 0.001 (***)
2.1 to 4	0.0230 (*)
4.1 to 6	0.0606 (ns)
above 6	0.02761 (*)

Supplemental Table 3 Statistical significance values for Figure 5l (two-way Anova with post hoc Tukey)

G1 phase

Samples	Significance
Ctl vs. Ctl ox	<0.001 (***)
Ctl vs. p107KO	<0.001 (***)
Ctl vs. p107KO ox	<0.001 (***)
Ctl ox vs. p107KO	<0.001 (***)
Ctl vs. p107KO ox	<0.001 (***)
p107KO vs. p107KO ox	Non-significant

S phase

Samples	Significance
Ctl vs. Ctl ox	<0.001 (***)
Ctl vs. p107KO	0.0089 (**)
Ctl vs. p107KO ox	0.0043 (**)
Ctl ox vs. p107KO	<0.001 (***)
Ctl vs. p107KO ox	<0.001 (***)
p107KO vs. p107KO ox	Non-significant

G2 phase

Samples	Significance
Ctl vs. Ctl ox	Non-significant
Ctl vs. p107KO	<0.001 (***)
Ctl vs. p107KO ox	<0.001 (***)
Ctl ox vs. p107KO	0.0044 (**)
Ctl vs. p107KO ox	<0.001 (***)
p107KO vs. p107KO ox	Non-significant

Supplemental Table 4 Primer Sets Used

Gene Name	Sequence Accession Number	Amplicon Length (bp)	Forward primer sequence	Reverse primer sequence
Rplp0 (36B4)	MGI:1927636	29	GAGGAATCAGATGAGG ATATGGGA	AAGCAGGCTGACTTGG TTGC
mt-Nd2 (Nd2)	MGI:102500	121	CATAGGGGCATGAGGA GGACT	TGAGTAGAGTGAGGGA TGGGTTG
mt-Nd6 (Nd6)	MGI:102495	44	TGTTGCAGTTATGTTGG AAGGAG	CAAAGATCACCCAGCT ACTACC
mt-Co2 (Cox2)	MGI:102503	98	AGTTGATAACCGAGTC GTTCTG	CTGTTGCTTGATTTAGT CGGC
mt-Atp6 (Atp6)	MGI:99927	55	TCCCAATCGTTGTAGC CATC	TGTTGGAAAGAATGGA GTCGG
D-loop	MF 133498.1	173	GCGTTATCGCCTCATA CGTT	GGTGCGTCTAGACTGT GTG
Nfe2l2 (Nrf2)	MGI:108420	146	AGAGCAACTCCAGAAG GAACAG	TGTGGGCAACCTGGGA GTAG
Mfn2	MGI:2442230	103	AGAGGCAGTTTGAGGA GTGC	ATGATGAGACGAACGG CCTC
Ppargc1a (Pgc-1α)	MGI:1342774	126	TACGCAGGTCGAACGA AACT	ACTTGCTCTTGGTGGA AGCA
Nrf1	MGI:1332235	154	GTTGGTACAGGGGCAA CAGT	TCGTCTGGATGGTCATT TCA
H19	MGI:95891	207	GTACCCACCTGTCGTC C	GTCCACGAGACCAATG ACTG
Mt-Co1	MGI:102504	342	CCCAATCTCTACCAGC ATC	GGCTCATAGTATAGCT GGAG
Slc25a4 (ANT 1)	MGI:1353495	174	GTCTCTGTCCAGGGCA TCAT	ACGACGAACAGTGTCA AACG
Rbl1 (p107)	MGI:103300	101	TCATTTGTGTTGGGCAC AGT	TACACCCAGGGGAAAC TCAG

Supplemental Materials and Methods

Mice

Housing, husbandry, and all experimental protocols for mice used in this study were performed in accordance with the guidelines established by the York University Animal Care Committee, which is based on the guidelines of the Canadian Council on Animal Care. The animal use protocols were approved by the Animal Care Committee of York University. Wild type and p107KO mice from Dr. M. Rudnicki are a mixed strain (NMRI, C57/Bl6, FVB/N) background. The mice were maintained in a temperature-and humidity-controlled 12-hr light-dark cycle. Food and water were provided ad libitum. Eight-to-10-week aged mice were used for derivation of prMPs and tissue immunofluorescence.

Cells

The c2c12 myogenic progenitor cell line (c2MP) was purchased from the American Tissue Type Culture (ATTC) and grown in Dulbecco's Modified Eagle Medium (DMEM) (Wisent) containing 25mM glucose supplemented with 10% fetal bovine serum (FBS) and 1% penicillin streptomycin. Primary myogenic progenitor cells (prMPs) were grown on rat tail collagen I (ThermoFisher Scientific) coated dishes containing Ham's F10 Nutrient Mix Media (ThermoFisher Scientific) supplemented with 20% FBS, 1% penicillin streptomycin and 2.5ng/ml bFGF (PeproTech).

For the nutrient specific experiments, c2MPs or prMPs were grown in stripped DMEM with 10% FBS and 1% penicillin streptomycin supplemented for 20 hours with 1mM, 5.5mM or 25mM glucose; or 4mM or 20mM glutamine; or for 6 hours with 4mM glutamine and 25mM glucose or 10mM galactose. For drug specific treatment, c2MPs or p107KO c2MPs were grown in DMEM with 10% FBS, 1% penicillin streptomycin, supplemented with 2.5mM oxamate (ox) (Sigma Aldrich) for 40 hours or p107KO c2MPs for 24 hours with 5mM metformin (TCI America). For

Sirt1 inhibition, cells were grown in stripped DMEM with 10% FBS,1% penicillin streptomycin and 5.5mM glucose with or without 10mM nicotinamide (nam) (Alfa Aesar) for 20 hours. For Sirt1 activation, cells were grown in 25mM DMEM with 10% FBS, 1% penicillin streptomycin with or without 1µM srt1720 (Selleckchem Chemicals) for 3 hours or 10µM resveratrol (res) (Santa Cruz Biotech) for 18 hours and for inactivation with 25µM res for 18 hours.

Cell synchronization

50,000 c2MPs were plated and cultured for 24 hours in DMEM supplied with 10% FBS, 1% penicillin streptomycin, before cell synchronization methods were applied to arrest the cells at G1 and G2 phases of cell cycle. To arrest c2MPs in G1, cells were washed twice with PBS grown in DMEM containing 1% FBS and 1% penicillin streptomycin for 72 hours. For G2 arrest, c2MPs were washed twice with PBS, refed and treated with 50ng/ml Nocodazole (Selleckchem Chemicals) for 18 hours.

Primary myogenic progenitor cell (prMP) isolation

All animal experiments were performed following the guidelines approved by the Animal Care Committee of York University. For derivation of prMPs and tissue immunofluorescence wild type and p107KO mice were from M. Rudnicki[1] maintained on a mixed (NMRI, C57/Bl6, FVB/N) background[2]. Extensor digitorum longus muscles of 3-month aged mice were dissected from tendon to tendon and digested in filter sterilized 0.2% type 1 collagenase (Sigma Aldrich) in serum free DMEM for 30 minutes. Upon unravelling, it was transferred to a pre-warmed petri dish containing DMEM (Wisent) with 1% penicillin, streptomycin. The muscle was then flushed gently with the media until it released the fibers with an intermittent incubation at 37°C every 5 minutes. After 30 minutes, individual fibers were transferred into 24 well tissue culture plate containing pre-warmed Ham's F10 Nutrient Mix Media (ThermoFisher Scientific) with 20% FBS, 1%

penicillin, streptomycin and 2.5ng/ml bFGF (PeproTech). After 3 days, the fibers were transferred to rat tail collagen I (ThermoFisher Scientific) coated tissue culture plates containing Ham's F10 Nutrient Mix Media with 20% FBS, 1% penicillin, streptomycin and 2.5ng/ml bFGF (PeproTech). The media was changed every alternate day, until the fibers generated prMPs that were collected by trypsinization and passaging onto collagen coated plates to remove live fibers.

Cloning

The p107mls expression plasmid that expresses p107 only in the mitochondria was made by cloning full length p107 into the pCMV6-OCT-HA-eGFP expression plasmid vector[3] that contains a mitochondrial localization signal. We used the following forward 5'-CACCAATTGATGTTCGAGGACAAGCCCCAC-3' and reverse 5'-CACAAGCTTTTAATGATTTGCTCTTTCACT-3' primer sets that contain the restriction sites Mfe1 and HindIII, respectively, to amplify full length p107 insert from a p107 Ha tagged plasmid[4]. The restriction enzyme digested full length p107 insert was then ligated to an EcoRI/HindIII digest of pCMV6-OCT-HA-eGFP, which removed the HA-eGFP sequences but retained the n-terminal mitochondrial localization signal (OCT).

Transfections

The calcium chloride method was used for most transfections, whereby a mixture containing a total of 10μg of plasmid DNA, 125mM $CaCl_2$ and H_2O were added dropwise to HEBS buffer (274mM NaCl, 10mM KCl, 1.4mM Na_2HPO_4, 15mM D-glucose and 42mM HEPES), incubated at room temperature for 1 hour and then added to 1×10^5 cells that had been passaged on the previous day. 18 hours post transfection, fresh DMEM containing 25mM glucose supplemented with 10% fetal bovine serum (FBS) and 1% penicillin streptomycin was added, and the cells used the next day. For overexpression studies, at least 4 different p107KO c2MPs were transfected as

above with GFP mitochondrial localization empty vector pCMV6-OCT-HA-eGFP[3], p107fl expressing full length p107 tagged HA, and p107mls expressing full length p107, which is directed to the mitochondria. For Sirt1 overexpression experiments, c2MPs were transfected with p107fl alone expressing full length p107 tagged HA or with full length (Sirt1fl) or dominant negative (Sirt1dn) Sirt1[5].

For generating Ldha knockdown cells, Lipofectamine 2000 (ThermoFisher Scientific) was used to transfect c2MPs with mouse Ldha siRNA ON-TARGETplus Smart pool (cat #L-043884-00-0005, Dharmacon) or mouse control siRNA ON-TARGETplus control pool (cat #D-001810-10-05) according to the manufacturer's protocol. For each transfection, 50nM of control or Ldha siRNA, or Lipofectamine 2000 were dissolved in two separate tubes with OptiMEM (ThermoFisher Scientific) and incubated for 5 minutes at room temperature. The tubes were then mixed and incubated for 20 minutes at room temperature before being added dropwise to the cells and incubated overnight at 37°C. The following day, the transfection media was changed to 25mM glucose DMEM with 10% serum. Cells were harvested 48 hours post transfection for assessments.

p107KO and SirtKO cell line derivation

Crispr/Cas9 was used to generate p107 and Sirt1 genetically deleted c2MP (p107KO and SirtKO) cell lines. For p107KO c2MPs, c2c12 cells were simultaneously transfected with 3 pLenti-U6-sgRNA-SFFV-Cas9-2A-Puro plasmids each containing a different sgRNA to target p107 sequences 110 CGTGAAGTCATCCAGGGCTT, 156 GGGAGAAGTTATACACTGGC and 350 AGTTTCGTGAGCGGATAGAA (Applied Biological Materials), and for Sirt1KO with 2 Double Nickase plasmids each containing a different sgRNA to target sequences 148 CGGACGAGCCGCTCCGCAAG and 110 CCATGGCGGCCGCCGCGGAA (Santa Cruz Biotechnology). Following 18 hours of incubation, the media was changed for 6 hours and then

passaged into 96 well tissue culture plates. On the next day, the media was aspirated and replenished with DMEM containing 2mg/ml puromycin for antibiotic selection. Following, the media was changed every 2 days until cell clones were visible. Any surviving clones was grown and tested by Western blotting. For control cells, c2MPs cells were transfected by empty pLenti-U6-sgRNA-SFFV-Cas9-2A-Puro (Applied Biological Materials) and selected as above.

Mitochondrial, nuclear and cytosolic isolation

For nuclear and cytoplasmic isolation, at least 1 million cells were pelleted, washed in PBS, dissolved in 500µl of cytoplasmic buffer (10mM Tris pH 7.4, 10mM NaCl, 3mM $MgCl_2$, 0.5% NP-40 with 1mg/ml of each pepstatin, leupeptin and aprotinin protease inhibitors) and incubated on ice for 5 minutes followed by rocking on ice for 5 minutes. After centrifugation at 2500g for 5 minutes at 4ºC, the supernatant was stored as the "cytoplasmic fraction". The cell pellet that represents the "nuclear fraction" was then washed 8 times with the cytoplasmic buffer and lysed with nuclear lysis buffer containing 50mM Tris pH 7.4, 5mM $MgCl_2$, 0.1mM EDTA, 1mM dithiothreitol (DTT), 40% (wt/vol) glycerol and 0.15 unit/µl benzonase (Novagen).

For mitochondrial and cytosolic isolation, at least 1 million cells were washed in PBS, pelleted, dissolved in 5 times the packed volume with isolation buffer (0.25M Sucrose, 0.1% BSA, 0.2mM EDTA, 10mM HEPES, pH 7.4; with 1mg/ml of each pepstatin, leupeptin and aprotinin protease inhibitors and 1mg/ml of protein tyrosine phosphatase inhibitor sodium orthovanadate, transferred into a prechilled Dounce homogenizer and homogenized loose (5-6 times) and tight (5-6 times) on ice. The homogenate was transferred into an eppendorf tube and centrifuged at 1000g at 4ºC for 10 minutes. The supernatant was then centrifuged at 14000g for 15 min at 4ºC and the resulting supernatant was saved as "cytosolic fraction". The pellet representing the "mitochondrial fraction"

was washed twice and dissolved in 50µl of isolation buffer. The mitochondria were lysed by repeated freeze-thaw cycles (3 times each) on dry ice.

Mitochondria fractionation

Mitochondrial fractions were isolated using a hypotonic osmotic shock approach[6]. For this, cells were collected by centrifugation at 380g and the cell pellet resuspended in STE buffer (250mM sucrose, 5mM Tris pH 7.4 and 1mM EGTA), Dounce homogenized and centrifuged at 1000g for 3 mins to remove cell debris. The supernatant was then centrifuged at 10,000g for 10 mins to isolate pelleted mitochondria. The mitochondria were resuspended in STE buffer containing 250mM sucrose and centrifuged at 16000g for 10 minutes to isolate matrix (M) and inner membrane (IM) proteins and the outer membrane (OM) proteins in the pellet and supernatant, respectively. To obtain a pure mitochondria matrix protein (M) fraction the isolated mitochondria were resuspended in hypotonic STE buffer containing 25mM sucrose and centrifuged at 16000g for 10 mins. After centrifugation, the soluble inner membrane (IMS) and outer membrane (OM) protein fractions were present in the supernatant and the matrix protein fraction (M) in the pellet. To demonstrate mitochondria matrix veracity, 50µg/ml porcine trypsin (Promega) was added for 30 mins followed by 20µg/ml trypsin inhibitor aprotinin (Roche) for 10 minutes before centrifugation at 16000g for 10 mins. Only fractions containing M proteins were unavailable for trypsin digestion. As a control whole cell treatment of c2MPs with both buffers showed that trypsin treatment can digest p107.

Western blot analysis

For Western blot analysis, cells were lysed in RIPA buffer (0.5% NP-40, 0.1% sodium deoxycholate, 150mM NaCl, 50mM Tris-Cl pH 7.5, 5mM EDTA) or mitochondrial isolation buffer (0.25M sucrose, 0.1% BSA, 0.2mM EDTA, 10mM HEPES, pH 7.4) containing 1mg/ml of

each of pepstatin, aprotinin and leupeptin, sodium orthovanadate. Protein lysates were loaded on gradient gels (6-15%), 6%, 7.5% or 10% gels. Proteins were transferred using a wet transfer method onto a 0.45μm pore sized polyvinylidene difluoride membrane (Santa Cruz Biotechnology) at 4ºC for 80 minutes at 100V. The membranes were blocked for an hour at room temperature in 5% non-fat milk in Tris-Buffered saline (TBS-150mM NaCl and 50mM Tris base) containing 0.1% Tween-20 (TBST). The membranes were probed overnight at 4ºC with primary antibodies (listed below) diluted in 5% non-fat milk or 1% BSA in TBST. The membranes were then washed three times with TBST and secondary antibodies conjugated with horseradish peroxidase diluted in 5% non-fat milk in TBST were added for an hour at room temperature with gentle rocking. The membranes were then washed 3 times with TBST, 5 minutes each and TBS for 10 minutes before the proteins were visualized with chemiluminescence on photographic films. Protein levels were evaluated by densitometry using Image J software.

Antibodies used

**Antibodies**	**Source**	**Catalogue Number/ Identifier**	**Antibody Dilutions**
α-tubulin	Proteintech	66031-1-Ig	1:5000
Brdu	Santa Cruz Biotech	Brdu-MoBU-1	1:100
Cox4	Abcam	ab16056	1:1000
E2f4	Cell Signaling	E2F4-E3G2G	1:1000
E2f5	Santa Cruz Biotech	E2F5-C8	1:1000
Ha	Santa Cruz Biotech	Ha-tag-F7	1:100
histone H3	Santa Cruz Biotech	histone H3-C16	1:500
histone H3	Cell Signaling	histone H3-D1H2	1:1000

MyoD	Novus Biologicals	NBP1-54153	1:100
Pax7	Santa Cruz Biotech	Pax-7-EE8	1:75
p107	Proteintech	13354-1-AP	1:1500
p107	Santa Cruz Biotech	p107-C18	1:2000
p107	Santa Cruz Biotech	p107-SD9	1:100
p130	Cell Signaling	RBL2-D9T7M	1:1000
Rb	Cell Signaling	Rb-D20	1:1000
Sirt1	Cell Signaling	Sirt1-D1D7	1:1000
Sirt1	Santa Cruz Biotech	Sirt1-B7	1:1000
Tfam	Proteintech	22586-1-AP	1:1000
Ldha	Cell Signaling	2012S	1:1000
OXPHOS rodent WB antibody cocktail	Abcam	ab110413	1:1000
Goat Anti-Rabbit IgG (H+L) HRP Conjugate	BioRad	170-6515	1:5000
Goat Anti-Mouse IgG (H+L) HRP Conjugate	BioRad	170-6516	1:5000
Goat Anti-Rabbit IgG (H+L) Cross-Adsorbed secondary antibody, Alexa Fluor 594	Life Technologies	A11012	1:200
Donkey Anti-Mouse IgG Secondary	Novus Biologicals	NL009	1:200

Antibody NL 493 conjugated			

Co-immunoprecipitation

For Immunoprecipitation (IP), protein lysates were pre-cleared with 50µl protein A/G plus agarose beads (Santa Cruz Biotechnology) by rocking at 4ºC for an hour. The sample was centrifuged at 21000g for a minute. Fresh protein A/G agarose beads along with 5µg of p107-C18, Sirt1-B7 or IgG-D7 antibody (Santa Cruz Biotechnology) antibody were added to the supernatant and rocked overnight at 4ºC. The next day the pellets were washed 3 times with wash buffer (50mM HEPES pH 7.0, 250mM NaCl and 0.1% NP-40) and loaded onto polyacrylamide gels and Western blotted for p107-SD9 (Santa Cruz Biotechnology) or Sirt1-D1D7 (Cell Signaling). Inputs represent 10% of lysates that were immunoprecipitated.

For IP of cytoplasmic fractions, 40ul of PureProteome protein A/G mix magnetic beads (EMD Millipore Corp) were used and incubated with 10ug of antibody; p107-SD9, Sirt1-B7 or IgG-D7 (Santa Cruz Biotechnology) at 4ºC for 30 minutes while rocking. After, cytosolic lysates were added for incubation overnight at 4ºC while rocking. The following day the lysates were washed, loaded onto SDS-PAGE gels and Western blotted for p107 (13354-1-AP) or Sirt1 (D1D7). Inputs represent 10% of lysates that were immunoprecipitated.

p107 mitochondria and cytoplasm percent occupancy

To determine the percentage of p107 occupancy in total, mitochondrial and cytoplasmic fractions, 25% and 75% of $2x10^6$ c2MPs were used to generate whole cell and mitochondrial/cytoplasmic lysates respectively. The lysates were loaded onto polyacrylamide gels and Western blotted for p107 (13354-1-AP, Proteintech), α-tubulin (66031-1-Ig, Proteintech) and Cox4 (ab16056,

Abcam). The ratio of p107:Cox4 and p107: α-tubulin was determined for whole cell and mitochondrial or cytoplasmic protein lysates, respectively, by densitometry using the ImageJ software. Then the percent of mitochondrial or cytoplasmic p107 occupancy was ascertained as a percentage of their ratios against the whole cell fraction ratio.

p107 internal mitochondria targeting signal-like (iMTS-L) sequence prediction

The p107 protein sequences that were analyzed for a potential iMTS-L sequence were the polypeptides resulting from a sequential removal of a single amino acid from the N terminus end of p107[7, 8, 9]. The resulting polypeptides were analyzed for the presence of an iMTS-L sequence using the TargetP prediction algorithm with the options set to non-plant organism, without cut-off and cleavage site prediction[9]. The resulting mitochondrial targeting peptide probability (mTP) values obtained from the TargetP algorithm were plotted against the corresponding amino acid. A threshold mTP value of 0.75 was used to define the presence of a potential iMTS sequence[7, 8, 9].

qPCR

RNA was isolated using Qiazol reagent (Qiagen) and concentration was determined by NanoDrop 2000 (ThermoFisher Scientific). qPCR experiments were performed according to the MIQE (Minimum Information for Publication of Quantitative Real-Time PCR Experiments) guidelines [10]. The optical density (OD) of RNA was measured using the NanoDrop 2000 (Thermo Fisher Scientific), RNA purity was inferred by the A260/280 ratio (~1.80 is pure). 1μg of RNA was reverse transcribed into cDNA using the All-in-One cDNA Synthesis SuperMix (Bimake) and the cDNA used for qPCR. qPCR assays were performed on Light cycler 96 (Roche) using SYBR green Fast qPCR Master mix (Bimake) with appropriate primer sets and Rplp0 (36B4) as a normalization control was used (Supplemental Table 4). Relative expression of cDNAs was determined with 36B4 as the internal control using the ΔΔCt method. For fold change, the ΔΔCt

was normalized to the control. Student t-tests, one-way or two-way Anova and Tukey post hoc tests were used for comparison and to obtain statistics.

Mitochondrial and nuclear DNA content

To obtain the relative mtDNA copy number to nDNA (mtDNA/nDNA), cells grown on a 6cm tissue culture plate were untreated or treated with 1mM 5-aminoimidazole-4-carboxamide-1-β-D-ribofuranoside (AICAR) (Toronto Research Chemicals) for 24hrs in presence of 5.5mM or 25mM glucose, with or without 10mM nam or 1µM srt1720. The cells were washed in PBS and lysed with 600µl of lysis buffer containing 100mM NaCl, 10mM EDTA, 0.5% SDS solution, 20mM Tris HCl; pH 7.4 and 6µl of 20mg/ml proteinase k (ThermoFisher Scientific). Following incubation at 55ºC for 3 hours, 100µg/ml RNase A (ThermoFisher Scientific) was added at 37ºC for 30 minutes. After, 250µl of 7.5M ammonium acetate and 600µl of isopropanol were added and the cells were centrifuged at 15000g for 10 minutes at 4ºC. The supernatant was discarded and the pellet containing mitochondrial and nuclear DNA was washed with 70% ethanol and resuspended in Tris EDTA buffer (10mM Tris HCl pH 7.4, 1mM EDTA). qPCR assays were performed on 50ng DNA with primer sets to Mt-Co1 and H19 (Supplemental Table 4) representing total mitochondrial and nuclear DNA content, respectively. Ct values were obtained and the ratio mtDNA:nDNA were determined by the formula: $2*2^{(nDNA\ Ct- mtDNA)}$ Ct.

Quantitative chromatin immunoprecipitation assay (qChIP)

qChIP was performed on mitochondrial lysates containing only mitochondrial DNA. Mitochondrial fractions were collected as described above, washed twice in PBS by centrifugation at 14000g for 15 minutes at 4°C, resuspended in 200µl of PBS containing 1% formaldehyde and rocked at room temperature for 30 minutes to fix the cells. The fixation reaction was quenched by adding 125mM of glycine in PBS and rocked for 5 minutes at room temperature. The fixed pellet

was washed twice in PBS containing 100mM NaF and 1mM Na_3VO_4 by centrifuging at 14000g for 5 minutes at 4°C. The pellet was the resuspended in 500µl of ChIP lysis buffer (40mM Tris, pH 8.0, 1% Triton X-100, 4mM EDTA, 300mM NaCl) and sonicated at 24% amplitude, 15 seconds on, 15 second off for 3 cycles (Model 120 Sonic Dismembrator, ThermoFisher Scientific). Following sonication, the samples were centrifuged at 16000g for 10 minutes at 4°C and the supernatant was transferred to a new tube containing 200µl of dilution buffer 1 (40mM Tris, pH 8.0, 4mM EDTA) from which the input controls were removed before 300 µl of dilution buffer 2 (40mM Tris, pH 8.0, 0.5% Triton X-100, 4mM EDTA, 150mM NaCl) was added. To preclear, 50µl of protein A/G agarose beads (Santa Cruz Biotechnology) was added and rocked for 90 minutes at 4°C. The beads were then pelleted and discarded, and to the supernatant was added 5µg of p107 antibody (p107- C-18) (Santa Cruz Biotechnology), E2f4 (E3G2G, Cell Signaling), Tfam (22586-1-AP, Proteintech) or IgG antibody (IgG-D-7) (Santa Cruz Biotechnology). This was rocked overnight at 4°C and on the following day, 50µl of protein A/G agarose beads were added and rocked for 90 minutes at 4°C. The beads were collected by centrifugation and were washed sequentially with 5 minutes rocking at 4°C before centrifugation by adding the following: low salt complex wash buffer (0.1% SDS, 1% Triton X-100, 2mM EDTA, 20mM Tris HCl, pH 8.0, 150mM NaCl), high salt complex wash buffer (0.1% SDS, 1% Triton X-100, 2mM EDTA, 20mM Tris HCl, pH 8.0, 500mM NaCl), LiCl wash buffer (0.25M LiCl, 1% NP-40, 1% deoxycholic acid, 1mM EDTA, 10mM Tris, pH 8.0) and 2 washes with TE buffer (10mM Tris HCl, pH 8.0, 1mM EDTA). After the last wash, the mtDNA-protein complexes were isolated by resuspending the beads in 250µl of elution buffer (1% SDS, 0.1M $NaHCO_3$), vortexing, rocking for 15 minutes at room temperature and centrifuging at 400g in a microcentrifuge for 2 minutes. The supernatant was transferred to a clean tube and to the remaining beads another 250µl of elution buffer was

added and the isolation step repeated. For isolation of mtDNA fragments, to the 500μl of mtDNA-protein complexes in elution buffer, 20μl of 5M NaCl was added and incubated at 65°C overnight. The next day, the DNA was isolated using DNA purification kit (Qiagen), and the concentration determined using the NanoDrop 2000 (ThermoFisher Scientific). Relative occupancy was determined by the ΔCt method by amplifying isolated DNA fragments using the D-loop primer sets (Supplemental Table 4) to obtain Ct values that are normalized to IgG Ct values.

NAD^+/NADH assay

Cells growing in 6cm tissue culture plates were washed with PBS and scraped into 500μl of 0.5M perchloric acid, vortexed and freeze-thawed on dry ice three times. The cells were then centrifuged at 4°C for 5 minutes at 4600g in a microfuge and 100μl of 2.2M $KHCO_3$ was added to the supernatant on ice. This was again centrifuged at 4600g for 15 minutes at 4°C and the supernatant was collected to be analyzed. For the lactate assay; 20μl of the supernatant, 258μl of lactate buffer (1M glycine, 500mM hydrazine sulfate, 5mM EDTA), 20μl of 25mM β-nicotinamide-adenine dinucleotide (NAD) (Roche) and 2μl of porcine heart lactate dehydrogenase (Ldh) (Sigma-Aldrich) were added to each well of an assay plate. For the pyruvate assay; 20μl of the supernatant, 218μl of pyruvate buffer (1.5M Tris, pH 8), 180μl of 6μM β-nicotinamide-adenine dinucleotide, reduced (NADH) (Roche) and 2μl of rabbit skeletal muscle LDH (Sigma-Aldrich) were added for each well. The assay plates were then read by a microplate reader (Glomax, Promega) with excitation at 340nm and emission peak at 450nm and the concentration values were attained by comparing to standard curves. The standard curves were made with NADH (Roche) at 0, 2, 3, 4, 5, 6, 7, and 10mM in lactate buffer for the lactate assay and in pyruvate buffer for the pyruvate assay. Estimation of free NAD^+/NADH in cells was based on lactate/pyruvate

(pyruvate + NADH + H^+ = lactate + NAD^+). The results were normalized to control samples and graphed.

ATP generation capacity and rate assay

The ATP generation capacity assay is based on the requirement of luciferase for ATP in producing light from the reaction:

$$\text{Luciferin} + \text{ATP} + O_2 \xrightarrow{\boxed{\text{Luciferase}}} \text{Oxyluciferin} + \text{AMP} + \text{Pyrophosphate} + CO_2 + \text{Light}$$

It was measured from mitochondrial fractions by using the ATP determination kit (ThermoFisher Scientific). In each well of a 96 well assay plate, 10µl of the isolated mitochondria was added to 4µl of 100mM ADP and 86 µl of reaction mix (1.78µl ddH_2O, 100µl of 20X reaction buffer, 20µl of 0.1M DTT, 100µl of 10mM D-luciferin and 0.5µl firefly luciferase from a 5mg/mL stock solution, all supplied by the kit manufacturer). The plate was then read over a period of 15 minutes every 1 minute for emission peak at 560 nm by a microplate reader (Glomax, Promega). The amount of ATP generated was normalized to the protein content of the mitochondrial fractions determined by Bradford Assay Kit (Biobasic) and graphed over time. The ATP generation rate was calculated from the slope of the graph of ATP that was produced over time up to the point where its production reached a steady state.

Immunocytochemistry and confocal imaging

For confocal microscopy, cells were grown on Nunc Lab-Tek™ II chambered tissue culture plates (ThermoFisher Scientific), fixed for 5 minutes with 95% methanol and permeabilized for 30 minutes at 4ºC with blocking buffer (3% BSA and 0.1% saponin in PBS). For immunofluorescence during proliferation and growth arrest antigen retrieval was used by fixing in 4% paraformaldehyde for 10 minutes, followed by incubation with 2N HCl for 20 minutes at room temperature. The cells

were then neutralized with sodium citrate buffer (10mM sodium citrate, 0.05% Tween 20, pH 6.0) for 10 minutes before blocking buffer was added. After blocking, cells were incubated with primary antibody p107-SD9 or HA-tag-F7 (Santa Cruz Biotechnology) in 1:100 dilution in blocking buffer for 1 hour. After 3 washes with 0.05% saponin in PBS (SP), cells were incubated with secondary antibody donkey anti-mouse IgG NL493-conjugated (R and D Systems) at a 1:200 dilution in blocking buffer. Cells were then washed 3 times in SP and re-incubated with primary antibody Cox4 (Abcam) at a 1:100 dilution in blocking buffer for 1 hour. After 3 washes in SP, cells were incubated with secondary antibody goat anti rabbit IgG Alexa Fluor 594 (ThermoFisher Scientific) in 1:200 dilution for 1 hour. After washing 3 times with SP, 4′,6-diamidino-2-phenylindole (Dapi) and Vectashield mounting media (Vector) was added before placing the coverslip. Confocal images and Z-stacks were obtained using the Axio Observer.Z1 microscope with alpha Plan-Apochromat 63x/Oil DIC (UV) M27 (Zeiss). Digital images were captured using Axiocam MR R3 (Zeiss). Optical sections were then "stacked" or merged to create high resolution "z-series" images. Z-stack images were also portrayed as orthogonally projected on XY, YZ and XZ plane with maximum intensity using ZEN imaging software (Zeiss). A line was drawn through a representative cell to indicate relative intensity of RGB signals.

For immunostaining with MitoTracker red, c2MPs were washed with PBS twice, fixed for 10 minutes with 95% methanol and permeabilized for 1 hour at room temperature in blocking buffer (3% BSA and 0.1% saponin in PBS). Then the cells were incubated with 500nM MitoTracker (Deep Red FM, Invitrogen) for 1 hour at room temperature. Cells were washed twice with PBS and incubated with primary antibody p107-SD9 in 1:100 dilution in blocking buffer for 1 hour. After 3 washes with SP, cells were incubated with secondary antibody donkey anti-mouse IgG NL493-conjugated at a 1:200 dilution in blocking buffer. After washing 3 times with SP, Dapi and

Vectashield mounting media (Vector) were added before placing the coverslip. Confocal images and Z-stacks were obtained using the Axio Observer.Z1 microscope with alpha Plan-Apochromat 63x/Oil DIC (UV) M27 (Zeiss).

Mitochondrial length and area measurement

Live cell imaging was used to measure mitochondrial length and area of **c**ells grown on 35mm high glass-bottom μ-Dish tissue culture plates (MatTek Corp). The cells were washed with PBS and stained with 1μl of MitoView red (Biotium) in 5ml serum free DMEM for 30 minutes. The cells were then washed with PBS and refed with serum free DMEM and immediately live cell imaged using the Axio Observer.Z1 (Zeiss) microscope with alpha plan-apochromat 40x/Oil DIC (UV) M27 (Zeiss) in an environment chamber (5% CO_2; 37°C). Digital images were captured using Axiocam MR R3 (Zeiss). Mitochondrial length was measured by tracing the mitochondria from one end to the other with a line that was calibrated to the scale bar using Image J software. Area was measured using Image J software according to Ouellet et al [11]. Briefly, threshold was used to select the area of each mitochondrian. The background was eliminated and using 'analyze particle', the area of each mitochondrian was measured by considering the scale bar as the reference point. At least 100 mitochondria were measured for length and area from each of 3 different controls (Ctl) and p107KO c2MP cell lines. The measurements were categorized and graphed within different intervals of lengths and areas, respectively.

Cardiotoxin-induced muscle regeneration

Three-month-old anesthetized wild type and p107KO mice were injected intramuscularly in the tibialis anterior (TA) muscle with 40μl of cardiotoxin (ctx) Latoxan (Sigma) that was prepared by dissolving in water to a final concentration of 10μM. A day after ctx injury, bromodeoxyuridine (Brdu) at 100mg/kg was injected intraperitonially. TA muscles were collected on day 2 post ctx

injection, immersed in a 1:2 ratio of 30% sucrose:optimal cutting temperature compound (ThermoFisher Scientific) solution and frozen slowly in liquid nitrogen-cooled isopentane. Mice were also untreated or treated with 750mg/kg ox for four consecutive days, with ctx on the third day and brdu on the fourth day, before the TA muscles were dissected on the fifth day for freezing.

Immunohistochemistry

The frozen samples were cross sectioned at 10µm thickness on a cryostat and mounted on positive charged slides (FroggaBio). Muscle tissue sections were washed with PBS and fixed with 4% paraformaldehyde (PFA) for 15 minutes at room temperature. 2N HCl was added for 20 minutes followed by 40mM sodium citrate buffer for 10 minutes at room temperature. After washing in PBS, muscle sections were blocked in blocking buffer (5% goat serum, 0.1% Triton X in PBS) for 30 minutes. Muscle tissue sections were then incubated with primary antibody anti-MyoD (Novus Biologicals) in blocking buffer in 1:100 ratio overnight or p107-SD9 or Pax7-EE8 in blocking buffer for 90 minutes. After three washes in 0.05% Tween 20 in PBS (PBST), cells were incubated with secondary antibody goat anti rabbit IgG Alexa Fluor 594 (ThermoFisher Scientific) or donkey anti-mouse IgG NL493-conjugated (R and D Systems) for one hour in 1:200 ratio. After three washes in PBST the tissue section was re-incubated with primary antibody Brdu-MoBU-1 (Santa Cruz Biotechnology) or Cox4 (Abcam) in blocking buffer in 1:100 ratio for 1 hour. After washing 3 times in PBST, the tissue section was incubated with secondary antibody donkey anti-mouse IgG NL493-conjugated (R and D Systems) or goat anti rabbit IgG Alexa Fluor 594 (ThermoFisher Scientific) in 1:200 ratio for 1 hour. After washing 3 times in PBST, Dapi was added and Vectashield mounting media (Vector) was added before placement of coverslip. The sample was imaged using confocal microscopy with the Axio Observer.Z1 microscope with alpha Plan-Apochromat 40x/Oil DIC (UV) M27 (Zeiss). Pax7 positive proliferating MPs containing p107 co-

localized with Cox4 were identified in successive tissue sections that were cut at 6μm instead of 10μm. Proliferating MPs were determined by enumerating the $MyoD^+Brdu^+Dapi^+$ cells as a percentage of all the $Dapi^+$ cells per field. Six fields per muscle section from 4 to 5 different mice for each treatment was used for the analysis.

Growth curve and Proliferation Rate

3000 cells were plated for both control and p107KO c2MPs that were untreated or treated with 2.5mM ox or 5mM metformin for 3 days. On each of the following 3 days, the number of cells were counted and graphed. Proliferation rate was calculated from the slope of the cell proliferation over time.

Flow Cytometry

For cell cycle analysis 50000, Ctl and p107KO cells were treated or untreated with 2.5mM ox for 40hrs or c2MPs were grown in 5.5mM or 25mM glucose in stripped or complete media for 20 hours or Ctl and Sirt1 KO cells were grown in 5.5mM glucose (which normally activate Sirt1), for 20 hours or p107KO cells 24 hours post transfection with pCMV6-OCT-HA-eGFP alone or together with p107fl or p107mls or p107KO cells untreated or treated with 5mM metformin for 24 hours or c2MPs synchronized at G1 and G2 phases of cell cycle. The cells were washed twice with PBS by centrifuging at 1200g for 5 minutes and re-dissolved in 1ml of PBS. Cells were then fixed by adding the cell suspension dropwise to 9ml of 70% ethanol while vortexing and then kept at -20°C overnight. The next day, the fixed cells were pelleted at 1500g for 5 minutes and resuspended with 3ml PBS on ice for 10 minutes to rehydrate the cells. The rehydrated cells were centrifuged at 1500g for 5 minutes and dissolved in 500μl of PBS containing 50μg/ml propidium iodide (ThermoFisher Scientific) and 25μg/ml RNAse (ThermoFisher Scientific) for at least 2hrs before loading on the Attune Nxt Flow Cytometer (ThermoFisher Scientific). Forward and side scatter

were appropriately adjusted and propidium iodide was excited with the 488-nm laser and detected in the BL2 channel. The excitation was analyzed by the ModFit LT™ software that provided the percentage of cells present in each of G1, S and G2 phases of cell cycle, which were represented graphically. Flow cytometry for cell cycle analysis of transfected cells consisted of cells transfected with empty vector pCMV6-OCT-HA-eGFP expressing GFP alone or along with p107fl expressing full length p107 or with p107mls expressing full length p107, which is directed to the mitochondria. GFP positive cells were first sorted using the BL1 channel of the cytometer by adjusting forward and side scatter, followed by detection of propidium iodide that was excited with the 488-nm laser and detected in the BL2 channel. The excitation was analyzed by the ModFit LT™ software that provided the percentage of cells present in each of G1, S and G2 phases of cell cycle and represented graphically.

Live cell ATP analysis (Seahorse)

3000 cells were seeded in DMEM (Wisent) containing 10% FBS and 1% penicillin streptomycin on microplates (Agilent Technologies) and treated according to the required experiment. The cells that were used were control or p107KO cells or p107KO cells transfected with pCMV6-OCT-HA-eGFP alone or together with p107fl or p107mls or p107KO cells untreated or treated with 5mM metformin or c2MPs and Sirt1KO c2MPs untreated or treated with 1µM srt1720. On the day of analysis, cells were washed in XF assay media supplemented with 10mM glucose, 1mM pyruvate and 2mM glutamine (Agilent Technologies) and incubated in a CO_2 free incubator in the same media at 37°C for 1 hour. The media was then removed and replenished with fresh XF media supplemented with 10mM glucose, 1mM pyruvate and 2mM glutamine and the microplate was assessed using the Seahorse XF real-time ATP rate assay kit (Agilent Technologies) on a calibrated Seahorse XFe96 extracellular flux analyzer (Agilent Technologies), with addition of 1.5µM of

oligomycin and 0.5μM rotenone plus antimycin A as per the manufacturer's direction. The energy flux data in real time was determined using Wave 2.6 software.

Statistics and Reproducibility

No statistical method was used to pre-determine sample size. All experiments were performed with at least three biological replicates as indicated in the figure legends, and results are presented as the mean ± standard deviation (SD). The immunoblot (Fig. 1a, c, d, n, 2b, g, 4b, e, k and supplementary figures 5a, b, 13a, 18a, 19a), co-immunoprecipitation (Fig. 4A and supplementary figure 15) and immunostaining (Fig. 1e, g, h, j, l, and supplementary figures 1; 4a, c, d, f, 6, 20, 21, 23, 24a, 30) experiments have been performed at least three independent times with similar results. Experimental design incorporated user blinding when possible. Statistical analysis was performed using GraphPad Prism. Statistical comparisons between groups were made using two-tailed unpaired Student's T test or an appropriate one-way or two-way analysis of variance (ANOVA) with a criterion of $p < 0.05$. All significant differences for ANOVA testing were evaluated using a Tukey post hoc test. Results were considered statistically significant when $p < 0.05$. The level of significance is indicated as follows: *$p < 0.05$, **$p < 0.01$, ***$p < 0.001$.

Supplementary References

1. LeCouter JE*, et al.* Strain-dependent myeloid hyperplasia, growth deficiency, and accelerated cell cycle in mice lacking the Rb-related p107 gene. *Mol Cell Biol* **18**, 7455-7465 (1998).

2. Chen D, Livne-bar I, Vanderluit JL, Slack RS, Agochiya M, Bremner R. Cell-specific effects of RB or RB/p107 loss on retinal development implicate an intrinsically death-resistant cell-of-origin in retinoblastoma. *Cancer Cell* **5**, 539-551 (2004).

3. Newman LE, Schiavon C, Kahn RA. Plasmids for variable expression of proteins targeted to the mitochondrial matrix or intermembrane space. *Cell Logist* **6**, e1247939 (2016).

4. Iwahori S, Umana AC, VanDeusen HR, Kalejta RF. Human cytomegalovirus-encoded viral cyclin-dependent kinase (v-CDK) UL97 phosphorylates and inactivates the retinoblastoma protein-related p107 and p130 proteins. *J Biol Chem* **292**, 6583-6599 (2017).

5. Boily G, He XH, Pearce B, Jardine K, McBurney MW. SirT1-null mice develop tumors at normal rates but are poorly protected by resveratrol. *Oncogene* **28**, 2882-2893 (2009).

6. Lu G, Sun H, Korge P, Koehler CM, Weiss JN, Wang Y. Functional characterization of a mitochondrial Ser/Thr protein phosphatase in cell death regulation. *Methods in enzymology* **457**, 255-273 (2009).

7. Backes S*, et al.* Tom70 enhances mitochondrial preprotein import efficiency by binding to internal targeting sequences. *J Cell Biol* **217**, 1369-1382 (2018).

8. Rahbani JF*, et al.* Creatine kinase B controls futile creatine cycling in thermogenic fat. *Nature* **590**, 480-485 (2021).

9. Emanuelsson O, Brunak S, von Heijne G, Nielsen H. Locating proteins in the cell using TargetP, SignalP and related tools. *Nat Protoc* **2**, 953-971 (2007).

10. Bustin SA*, et al.* The MIQE guidelines: minimum information for publication of quantitative real-time PCR experiments. *Clin Chem* **55**, 611-622 (2009).

11. Ouellet M, Guillebaud G, Gervais V, Lupien St-Pierre D, Germain M. A novel algorithm identifies stress-induced alterations in mitochondrial connectivity and inner membrane structure from confocal images. *PLoS Comput Biol* **13**, e1005612 (2017).
