# Supplementary material for: p107 mediated mitochondrial function controls muscle stem cell proliferative fates
Source: Nat Commun. 2021 Oct 13;12:5977. doi: 10.1038/s41467-021-26176-0 (PMC8514468; doi:10.1038/s41467-021-26176-0)
Supplement: Supplementary file 3 — Reporting Summary [file 41467_2021_26176_MOESM3_ESM.pdf]

Corresponding author(s): Anthony Scimè
Last updated by author(s): Aug 14, 2021

Reporting Summary

Nature Research wishes to improve the reproducibility of the work that we publish. This form provides structure for consistency and transparency in reporting. For further information on Nature Research policies, see our Editorial Policies and the Editorial Policy Checklist.

Statistics

For all statistical analyses, confirm that the following items are present in the figure legend, table legend, main text, or Methods section.

n/a	Confirmed	
☐	☒	The exact sample size (n) for each experimental group/condition, given as a discrete number and unit of measurement
☐	☒	A statement on whether measurements were taken from distinct samples or whether the same sample was measured repeatedly
☐	☒	The statistical test(s) used AND whether they are one- or two-sided<br>*Only common tests should be described solely by name; describe more complex techniques in the Methods section.*
☒	☐	A description of all covariates tested
☐	☒	A description of any assumptions or corrections, such as tests of normality and adjustment for multiple comparisons
☐	☒	A full description of the statistical parameters including central tendency (e.g. means) or other basic estimates (e.g. regression coefficient) AND variation (e.g. standard deviation) or associated estimates of uncertainty (e.g. confidence intervals)
☒	☐	For null hypothesis testing, the test statistic (e.g. F, t, r) with confidence intervals, effect sizes, degrees of freedom and P value noted<br>*Give P values as exact values whenever suitable.*
☒	☐	For Bayesian analysis, information on the choice of priors and Markov chain Monte Carlo settings
☒	☐	For hierarchical and complex designs, identification of the appropriate level for tests and full reporting of outcomes
☒	☐	Estimates of effect sizes (e.g. Cohen's d, Pearson's r), indicating how they were calculated

Our web collection on statistics for biologists contains articles on many of the points above.

Software and code

Policy information about availability of computer code

Data collection	Microscopy: Axio Observer.Z1 microscope with alpha Plan-Apochromat 63x/Oil DIC (UV) M27 and alpha Plan-Apochromat 40x/Oil DIC (UV) M27 (Zeiss), Flow Cytometry: Attune Nxt Flow Cytometer (Thermo Fisher), Metabolism: Seahorse XFe96 extracellular flux analyzer (Agilent Technologies), NAD+/NADH Assay: Glomax microplate reader (Promega), qPCR: Light cycler 96 (Roche)
Data analysis	Prism 9.2, ModFit LT (4.0), Zeiss Zen 3.1, Image J 1.52a, Target P 2.0

For manuscripts utilizing custom algorithms or software that are central to the research but not yet described in published literature, software must be made available to editors and reviewers. We strongly encourage code deposition in a community repository (e.g. GitHub). See the Nature Research guidelines for submitting code & software for further information.

Data

Policy information about availability of data

All manuscripts must include a data availability statement. This statement should provide the following information, where applicable:

- Accession codes, unique identifiers, or web links for publicly available datasets
- A list of figures that have associated raw data
- A description of any restrictions on data availability

The raw images for all the immuno blots and data sets for graphs are provided in the Source data file that accompanies this manuscript.

Field-specific reporting

Please select the one below that is the best fit for your research. If you are not sure, read the appropriate sections before making your selection.

☒ Life sciences ☐ Behavioural & social sciences ☐ Ecological, evolutionary & environmental sciences

For a reference copy of the document with all sections, see nature.com/documents/nr-reporting-summary-flat.pdf

Life sciences study design

All studies must disclose on these points even when the disclosure is negative.

Sample size	Sample size is indicated in the figure legends for each experiment. No sample size calculation was performed. Sample size was determined based on the magnitude and consistency of measurable differences between groups
Data exclusions	No data was excluded from analysis.
Replication	Biological and independent replicate experiments were successful. They were replicated independently by different co-authors.
Randomization	Wild type and p107KO mice were allocated to muscle regeneration experiments based on genotype and irrespective of sex. There were no other selection criteria for the allocated animals. Other experiments did not require randomization.
Blinding	The researchers were blinded to allocation during analysis and outcome assessment. Animal experiments were blinded when possible.

Reporting for specific materials, systems and methods

We require information from authors about some types of materials, experimental systems and methods used in many studies. Here, indicate whether each material, system or method listed is relevant to your study. If you are not sure if a list item applies to your research, read the appropriate section before selecting a response.

Materials & experimental systems

n/a	Involved in the study	
☐	☒	Antibodies
☐	☒	Eukaryotic cell lines
☒	☐	Palaeontology and archaeology
☐	☒	Animals and other organisms
☒	☐	Human research participants
☒	☐	Clinical data
☒	☐	Dual use research of concern

Methods

n/a	Involved in the study	
☒	☐	ChIP-seq
☐	☒	Flow cytometry
☒	☐	MRI-based neuroimaging

Antibodies

Antibodies used

α-tubulin Proteintech 66031-1-Ig 1:5000
Brdu Santa Cruz Biotech Brdu-MoBU-1 1:100
Cox4 Abcam ab16056 1:1000
E2f4 Cell Signaling E2F4-E3G2G 1:1000
E2f5 Santa Cruz Biotech E2F5-C8 1:1000
Ha Santa Cruz Biotech Ha-tag-F7 1:100
histone H3 Santa Cruz Biotech histone H3-C16 1:500
histone H3 Cell Signaling histone H3-D1H2 1:1000
MyoD Novus Biologicals NBP1-54153 1:100
Pax7 Santa Cruz Biotech Pax-7-EE8 1:75
p107 Proteintech 13354-1-AP 1:1500
p107 Santa Cruz Biotech p107-C18 1:2000
p107 Santa Cruz Biotech p107-SD9 1:100
p130 Cell Signaling RBL2-D9T7M 1:1000
Rb Cell Signaling Rb-D20 1:1000
Sirt1 Cell Signaling Sirt1-D1D7 1:1000
Sirt1 Santa Cruz Biotech Sirt1-B7 1:1000
Tfam Proteintech 22586-1-AP 1:1000
Ldha Cell Signaling 2012S 1:1000
OXPHOS rodent WB antibody cocktail Abcam ab110413 1:1000
Goat Anti-Rabbit IgG (H+L) HRP Conjugate BioRad 170-6515 1:5000
Goat Anti-Mouse IgG (H+L) HRP Conjugate BioRad 170-6516 1:5000

	Goat Anti-Rabbit IgG (H+L) Cross-Adsorbed secondary antibody, Alexa Fluor 594 Life Technologies A11012 1:200<br>Donkey Anti-Mouse IgG Secondary Antibody NL 493 conjugated Novus Biologicals NL009 1:200
Validation	The p107 and Sirt1 antibodies were verified by data provided in the manuscript . Other antibodies are verified from previous studies and the manufacturers listed in the Supplementary Materials and Methods.

Eukaryotic cell lines

Policy information about cell lines

Cell line source(s)	c2MPs (c2c12) were purcahsed from ATCC
Authentication	c2MP differentiation into myotubes when grown in 2% horse serum.
Mycoplasma contamination	The cells were not contaminated by mycoplasma as determined by the MycoSensor PCR Assay Kit (Agilent).
Commonly misidentified lines<br>(See ICLAC register)	No commonly misidentified cell lines were used in this study.

Animals and other organisms

Policy information about studies involving animals; ARRIVE guidelines recommended for reporting animal research

Laboratory animals	Wild type and p107KO mice were from Dr. M. Rudnicki in a mixed strain (NMRI, C57/Bl6, FVB/N) background. Maintained in a temperature-and humidity-controlled environment on a 12-hr light-dark cycle. Food and water were provided ad libitum. Bothe male and female 8 to 10 week aged mice were used for experiments.
Wild animals	This study did not involve wild animals.
Field-collected samples	This study did not involve animals collected in the field.
Ethics oversight	Housing, husbandry, and all experimental protocols for mice used in this study were performed in accordance with the guidelines established by the York University Animal Care Committee, which is based on the guidelines of the Canadian Council on Animal Care. The animal use protocols were approved by the Animal Care Committee of York University.

Note that full information on the approval of the study protocol must also be provided in the manuscript.

Flow Cytometry

Plots

Confirm that:

☒ The axis labels state the marker and fluorochrome used (e.g. CD4-FITC).

☒ The axis scales are clearly visible. Include numbers along axes only for bottom left plot of group (a 'group' is an analysis of identical markers).

☒ All plots are contour plots with outliers or pseudocolor plots.

☒ A numerical value for number of cells or percentage (with statistics) is provided.

Methodology

Sample preparation	Sample preparation using flow cytometry for cell cycle analysis can be found in the Methods section of the manuscript and Supplemental Materials and Methods.
Instrument	Attune Nxt Flow Cytometer (Thermo Fisher)
Software	FloJo 10.8
Cell population abundance	Purity of post-sort fractions was determined by flow cytometry on the sorted samples.
Gating strategy	The gating strategy is provided in Supplemental Figures 8 and 25 of this manuscript.

☒ Tick this box to confirm that a figure exemplifying the gating strategy is provided in the Supplementary Information.
